# Identification of small molecules and related targets that modulate tau pathology in a seeded primary neuron model

**DOI:** 10.1016/j.jbc.2023.104876

**Published:** 2023-06-01

**Authors:** Garrett S. Gibbons, Hailey Gould, Virginia M.-Y. Lee, Alex Crowe, Kurt R. Brunden

**Affiliations:** Department of Pathology and Laboratory Medicine, Center for Neurodegenerative Disease Research, Perelman School of Medicine, University of Pennsylvania, Philadelphia, Pennsylvania, USA

**Keywords:** amyloid-beta (Aβ), compound screening, neurodegeneration, neurons, pathology, tauopathy, tau protein, tau seeding, therapeutics

## Abstract

Alzheimer’s disease (AD) is characterized by the presence of tau protein inclusions and amyloid beta (Aβ) plaques in the brain, with Aβ peptides generated by cleavage of the amyloid precursor protein (APP) by BACE1 and γ-secretase. We previously described a primary rat neuron assay in which tau inclusions form from endogenous rat tau after seeding cells with insoluble tau isolated from the human AD brain. Here, we used this assay to screen an annotated library of ∼8700 biologically active small molecules for their ability to reduce immuno-stained neuronal tau inclusions. Compounds causing ≥30% inhibition of tau aggregates with <25% loss of DAPI-positive cell nuclei underwent further confirmation testing and assessment of neurotoxicity, and non-neurotoxic hits were subsequently analyzed for inhibitory activity in an orthogonal ELISA that quantified multimeric rat tau species. Of the 173 compounds meeting all criteria, a subset of 55 inhibitors underwent concentration-response testing and 46 elicited a concentration-dependent reduction of neuronal tau inclusions that were distinct from measures of toxicity. Among the confirmed inhibitors of tau pathology were BACE1 inhibitors, several of which, along with γ-secretase inhibitors/modulators, caused a concentration-dependent lowering of neuronal tau inclusions and a reduction of insoluble tau by immunoblotting, although they did not decrease soluble phosphorylated tau species. In conclusion, we have identified a diverse set of small molecules and related targets that reduce neuronal tau inclusions. Notably, these include BACE1 and γ-secretase inhibitors, suggesting that a cleavage product from a shared substrate, such as APP, might affect tau pathology.

A number of neurodegenerative diseases known as tauopathies are characterized by the presence of inclusions comprised of hyperphosphorylated tau protein fibrils within brain neurons (1, 2). These neuronal tau aggregates are referred to as neurofibrillary tangles when localized to the cell soma and neuropil threads when found in dendritic processes. The most prevalent of the tauopathies is Alzheimer’s disease (AD), and others include progressive supranuclear palsy, corticobasal degeneration, Pick’s disease, traumatic brain injury, chronic traumatic encephalopathy, and primary age-related tauopathy (2, 3, 4, 5). In addition to tau inclusions, a second hallmark pathology in AD are senile plaques comprised of amyloid β (Aβ) peptides (6). The prevailing “amyloid cascade hypothesis” (7, 8) posits that Aβ plaques, which can form up to 2 decades prior to the onset of AD symptomology, facilitate the spread of tau pathology from relatively confined limbic regions to higher cortical areas of the brain. The extent of cortical spread of tau pathology is highly correlated with the cognitive status of patients with AD (9, 10), and recent tau PET imaging studies further confirm that tau pathological burden is associated with both disease symptomology and brain atrophy (11, 12), suggesting that tau inclusions are the major source of the neuronal and synaptic damage seen in AD brain.

Tau is normally a microtubule (MT)-associated protein, where it appears to regulate MT dynamics (13, 14), accessibility of MT-severing enzymes (15) and the binding of motor proteins involved in axonal transport (16, 17). In AD and related tauopathies, tau becomes hyperphosphorylated through an imbalance of kinase and phosphatase function (1), resulting in enhanced disengagement of tau from MTs (18, 19, 20). This results in an increased cytosolic phospho-tau concentration that likely facilitates misfolding into the fibrillar species that comprise the hallmark tau inclusions. Given the aforementioned correlation between tau pathological burden and neurodegenerative outcomes in AD, there is a growing consensus that tau fibrils and/or pre-fibrillar oligomers are neurotoxic species.

The preponderance of AD drug discovery efforts has focused on strategies to lower Aβ plaque formation, largely motivated by evidence that familial AD can result from mutations in the amyloid precursor protein (APP) or the presenilin proteins found in the γ-secretase complex involved in the proteolytic cleavage of APP to yield Aβ peptides (21). However, the growing recognition that Aβ plaque deposition begins over a decade before symptomology, whereas tau pathological spread is linked to symptom onset, has led to growing recognition of the importance of developing pharmaceuticals to reduce tau inclusions. To date, the most abundant and clinically advanced tau-directed drug candidates have been tau monoclonal antibodies, which were developed with the hope they might slow the spread of tau pathology in the brain based on the emerging concept of “prion-like” transmission of pathology (22). Unfortunately, passive tau immunotherapy is yet to yield positive clinical results, with a number of potential explanations for the current lack of success (23, 24). Although there are additional tau drug discovery efforts ongoing at both the clinical and preclinical stages (25, 26), there would nonetheless be considerable value in identifying new drug targets and related small molecule modulators that would reduce the formation or persistence of misfolded tau species in AD and related tauopathies.

Tau-directed target identification and drug discovery efforts would be facilitated by unbiased phenotypic cellular assays of tau pathology formation that are amenable to medium-to-high throughput compound screening. In this regard, we recently described a primary rat neuron assay in which tau inclusions with similarities to neurofibrillary tangles and neuropil threads are formed from endogenous rat tau after seeding of the neurons with highly enriched insoluble tau isolated from human AD brain (27, 28). Importantly, this assay has been optimized for 384-well compound screening (27) and, unlike most previously described cell-based assays of tau inclusion formation (29, 30, 31, 32), it utilizes the cell type (neurons) most affected in tauopathies without transgene overexpression of tau. A prior limited screen of the Prestwick library of mostly approved drugs in this neuronal assay resulted in the identification of a number of compounds that showed concentration-dependent inhibition of tau inclusions, with many of these compounds directed to neuronal receptors (27).

Based on the promising results from our prior small screen, we describe here the screening in the neuronal tau inclusion assay of a larger library comprised of ∼8700 small molecules for which some understanding of the mechanism of action has been ascribed to each compound. Compounds that met prespecified hit criteria for reducing neuronal tau inclusions as assessed by immunofluorescence quantification of insoluble rat tau underwent secondary testing to eliminate compounds showing evidence of neurotoxicity. Non-neurotoxic confirmed hits were subsequently tested for activity in an orthogonal ELISA-based assay that quantifies multimeric tau species and a selected subset of compounds from the confirmed hit list were repurchased for concentration-response testing in the primary neuronal tau inclusion assay. As described below, these studies led to the identification of a diverse set of compounds and putative drug targets that affect tau inclusion formation in primary neurons, providing a valuable resource for further investigation of potential targets and small molecules that might ultimately lead to new drug discovery opportunities for the treatment of AD and related tauopathies. Among the confirmed inhibitors of neuronal tau pathology were compounds directed to BACE1 and γ-secretase, suggesting that one or more proteolytic fragments from a shared substrate such as APP may enhance tau inclusion formation.

## Results

### Assay optimization

**Figure 1:**
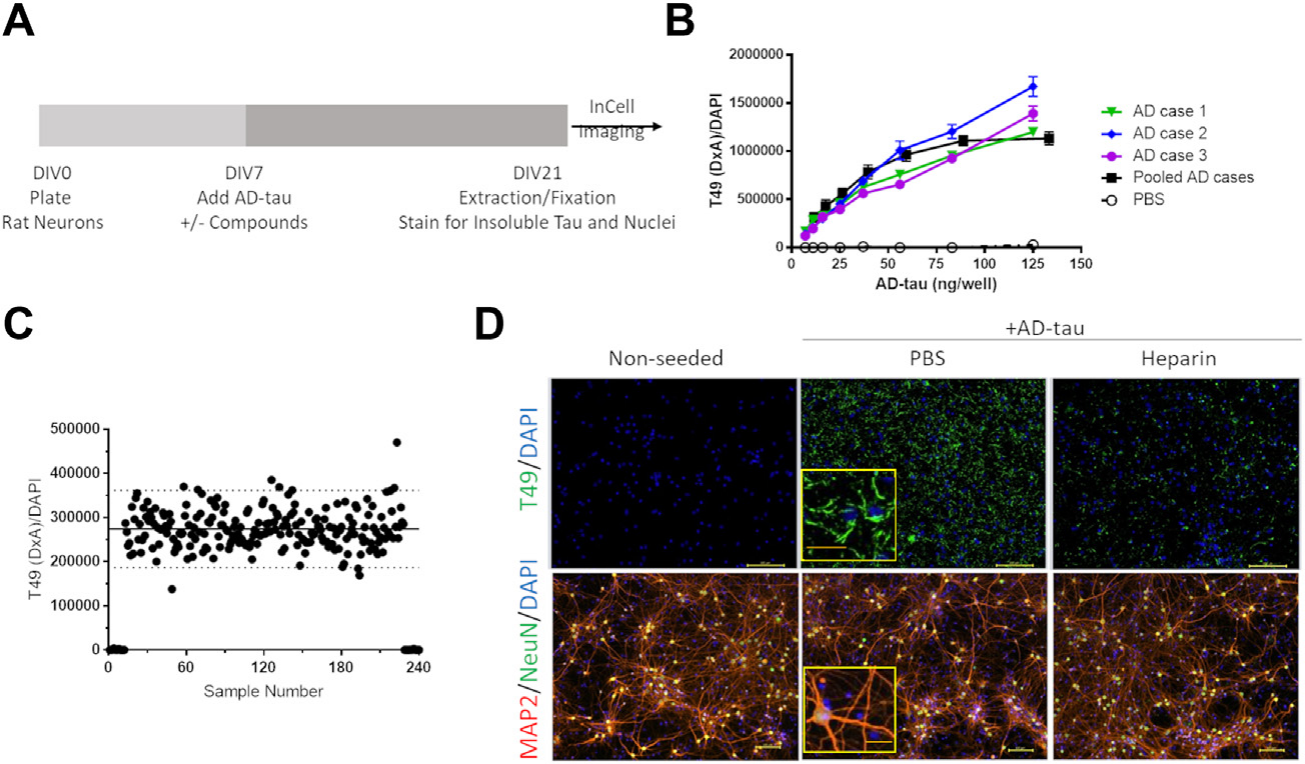
**Seeded aggregation of endogenous tau in primary rat neurons.***A*, schematic representation of the timeline of the primary rat cortical neuron assay of tau inclusion formation. Embryonic cortical neurons are plated at day *in vitro* 0 (DIV0), and brain-derived AD-tau and test compounds or vehicle are added at DIV7. At DIV21 the cultures are detergent-extracted and fixed prior to staining with the rodent tau-specific T49 antibody, with cell nuclei visualized *via* DAPI staining. The stained plates are then imaged, with quantification of integrated T49 staining signal and DAPI counts. *B*, assay dose–response curves of tau pathology induced in primary rat neurons from individual AD-tau case extractions as well as the pooled extracts. The amount of contaminating Aβ40/42 and α-synuclein in the pooled AD-tau preparation was determined by ELISA and was <0.1% and <0.25% of the tau concentration, respectively. *C*, quantification of tau inclusions across a representative 384-well plate. *Dashed lines* represent 3SD from the mean of AD-tau–treated wells. The samples near zero are wells that did not receive AD-tau. *D*, images of T49-stained rat tau pathology induced by AD-tau, and inhibition of tau seeding by heparin (3 mg/ml) co-treatment. The cultures were also stained with DAPI to visualize cell nuclei. MAP2 and NeuN staining in parallel cultures reveals the dendritic morphology and neuronal cell bodies, which are unaffected by AD-tau or heparin treatment. Scale bar = 0.1 mm; insets represent 4× magnification with scale bars = 0.4 mm.

The primary neuronal assay of tau inclusion formation used for compound screening has been described previously (27) and is depicted in Figure 1*A*. The key aspects of this assay that distinguish it from other reported cellular assays of tau aggregate formation are (1) the utilization of primary neurons, (2) the absence of human tau transgene expression, and (3) the initiation of tau inclusion formation through seeding of the neuron cultures with pathologic tau enriched from human AD brains (AD-tau) (28). Moreover, although the adult rodent brain contains tau with primarily four microtubule-binding repeats (4R) and thus differs from the human brain where both 4R and 3R tau are found in similar quantities, cultured rat neurons as utilized in our assay contain a mixture of both 4R and 3R rat tau (∼1:3 4R:3R ratio at DIV21). The combination of these features provides an assay with physiological relevance, normal neuronal tau expression levels, and tau pathology that is templated by human AD-tau fibrils.

To minimize variability during the compound screening endeavor, three separate preparations of AD-tau were generated from autopsied human brain with a neuropathological diagnosis of advanced AD (Braak stage 6), with representative immunohistochemical images of the extent of PHF1-positive tau pathology for each case depicted in Fig. S1*A*. The tau isolation method, as well as representative immunoblot and corresponding Ponceau S stained blot, are shown in Fig. S1, *B* and *C*, respectively. Notably, this isolation method yields highly enriched insoluble human tau that is nearly devoid of Aβ or α-synuclein contamination (*i.e.*, <0.25% Aβ:tau and α-synuclein:tau, w/w). Each of the three AD-tau preparations underwent dose-response testing in the primary neuronal assay, with insoluble tau pathology imaged after detergent extraction and subsequent fixation of the neurons, followed by immunofluorescence staining with the rodent tau-specific T49 antibody (*e.g.*, Fig. 1*D*) (27) to establish AD-tau doses that fell within the linear range of tau pathological seeding (Fig. 1*B*). As observed previously (27), the different AD-tau preparations showed nearly identical dose-dependent seeding profiles in the cortical neurons, and thus these separate preparations were pooled to provide a large AD-tau batch with activity comparable to the individual preparations (Fig. 1*B*) in sufficient quantity to support the compound screening effort.

Based on the dose-response activity profile of the pooled AD-tau preparation, 62.5 ng/well was chosen for compound screening, as this fell well within the linear range of response. The suitability of this AD-tau dose was confirmed through the analysis of multiple 384-well plates in which the majority of wells were seeded with AD-tau, with unseeded control wells also included allowing for the determination of the assay signal-to-background (*i.e.*, integrated T49 immunofluorescence signal normalized to DAPI counts in AD-tau treated *versus* untreated wells). Moreover, these test plates allowed for the determination of the assay Z′-score at this AD-tau dose, with a Z-score ≥0.5 indicating a highly suitable cell-based screening assay (33). The preliminary testing revealed a >2000-fold difference in normalized T49 fluorescent signal between the neurons seeded with AD-tau or those receiving only vehicle. Furthermore, appropriate Z′-scores were routinely achieved, and assessment of the number of wells that showed >3 standard deviations (SD) from the mean of AD-tau seeded wells revealed a low false-positive rate (typically <0.3%; example in Fig. 1*C*). Example images showing the extent of neuronal T49-positive tau aggregates in wells treated with AD-tau *versus* those that were not seeded are shown in Figure 1*D*, as are parallel AD-tau seeded and non-seeded cultures that were fixed and permeabilized followed by staining with NeuN (34) and MAP2 (35) antibodies to assess the number of neuronal cells and the dendritic process area, respectively. Finally, prior studies (36) have shown that the seeding of tau pathology by exogenous tau fibrils can be inhibited by heparin, and we confirmed that heparin could reduce the extent of AD-tau seeded pathology in the neuronal assay (Fig. 1*D*). Neurons receiving heparin in addition to AD-tau showed >60% inhibition of T49-positive tau inclusions at the non-toxic dose of 3 mg/ml, whereas greater heparin concentrations reduced cell viability.

Although the tau pathology that forms in the cultures has been demonstrated to co-localize with neuronal processes and soma, the cultures also contain non-neuronal cells. The predominant non-neuronal population is comprised of GFAP-positive astrocytes, which increase in numbers over time in culture such that they and neurons each represent approximately half of the cell population at the end of the 21 days of culture (Fig. S2). We could not detect a meaningful microglial population upon staining of the cultures with Iba1 antibody (not shown), but the lack of co-localization of some DAPI-stained nuclei with either NeuN-positive neurons or GFAP-positive astrocytes suggests there is also a small percentage of cells of an indeterminate type that we speculate may be endothelial cells and/or fibroblasts. Given the presence of non-neuronal cells in the assay, we cannot exclude the possibility that compounds might act to modulate neuronal tau inclusions through an indirect effect mediated by astrocytes or, less likely, other co-cultured cells.

### Compound screening, hit validation, and neurotoxicity assessment

To identify small molecule modulators of tau pathology, an annotated library with reported biological targets and/or pathways for each compound (MedChem Express Bioactive compound library) was screened in the primary neuron model of tau pathology. The compound library was diluted to 5 mM in DMSO and each compound was arrayed in triplicate in non-consecutive wells in 384-well plates, with a total of 60 compounds per plate. This resulted in a total of 148 384-well compound plates that were screened at 10 μM compound concentration (0.2% final DMSO) in the neuronal tau inclusion assay (summarized in Fig. 1*A*). Compounds were added to the neuron cultures at 7 days *in vitro* (DIV7) 1 h prior to the subsequent addition of AD-tau, and the cultures then underwent an additional 14-day incubation period without media exchange or compound re-addition. At DIV21, soluble tau was extracted through cationic detergent treatment and the cells were then fixed to allow assessment of tau pathology and cell numbers using the rodent tau-specific T49 antibody and DAPI immunofluorescence staining, respectively.

**Figure 2:**
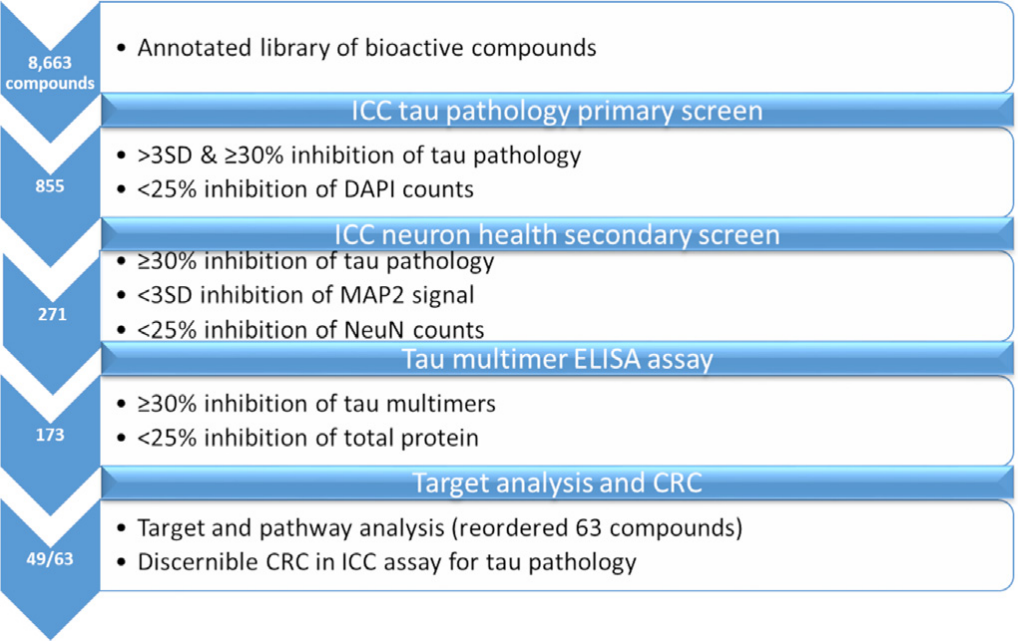
**Schematic workflow of compound screening and hit triage strategy, with the number of compounds successfully meeting the hit criteria at each step indicated in the associated chevron**.

The assay signal-to-background during the compound screening was again routinely >2000 and the well-to-well variability was low enough to provide a mean Z′-score of 0.61 for the entire screening endeavor (range 0.31–0.82). As compounds were screened in triplicate, a Z′-score of ≥0.4 was deemed acceptable, and plates not meeting this Z′-score criterion were repeated to achieve this Z′ threshold (8 of 148 plates). Upon completion of screening, a total of 855 compounds met the nominal hit definition of >3 standard deviation (SD) decrease and ≥30% total inhibition of tau pathology, with <25% reduction in total DAPI-positive cells per well (screening flow chart summary provided in Fig. 2). The hit threshold of 30% was chosen in addition to a >3SD decrease in tau inclusions to exclude compounds that met the latter threshold but showed a modest total reduction of tau aggregates (*e.g.*, in plates with a CV of <8%, 3SD would be <24% inhibition). Since the primary neuron cultures contain ∼50% non-neuronal cells, quantification of DAPI-positive cells provided a measure of total cellular toxicity, and a secondary analysis was subsequently conducted with the 855 hit compounds to more accurately assess the influence of compounds on neuron viability and health. The hits were formatted into new plates and were reanalyzed in the AD-tau-treated primary neuron cultures at 10 μM concentration as described earlier, except the cultures were now permeabilized and fixed with paraformaldehyde followed by staining for NeuN and MAP2 after the 21-day culture period to assess for adverse effects on neuron numbers or dendritic morphology, respectively. Most of the 855 hits were analyzed in triplicate, but because of a period of temporary constraints on available neurons, some compounds were tested in only a single well. Compounds were de-prioritized if they induced >25% reduction of NeuN-positive neurons or if MAP2 staining was reduced by >3SD compared to the mean of AD-tau-treated neurons receiving vehicle. The primary hits were also concurrently re-evaluated at 10 μM concentration in parallel cultures to confirm that they caused ≥30% inhibition of tau pathology. The combination of activity re-testing and determination of neuronal toxicity led to a total of 271 compounds with confirmed inhibitory activity of ≥30% and low neuronal toxicity (Fig. 2), with compounds eliminated due to evidence of decreased MAP2 and/or NeuN staining as well as failure to cause ≥30% inhibition of tau pathology in the repeat activity analysis.

### Testing of non-neurotoxic hits in an orthogonal tau multimer assay

**Figure 3:**
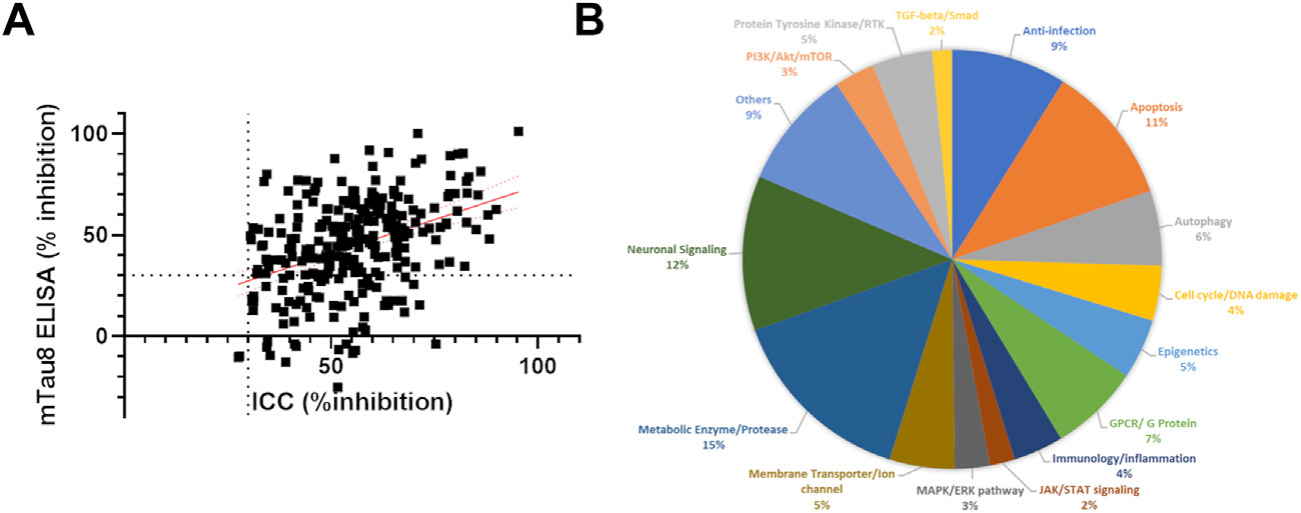
**Biochemical validation of screening hits and pathway analysis.***A*, the 271 compounds that caused ≥30% inhibition of neuronal tau inclusions without evidence of neurotoxicity were analyzed in a tau multimer ELISA, with 173 of the compounds also causing ≥30% inhibition of neuronal multimeric tau species (*upper right* quadrant). The line represents the linear fit (r^2^ = 0.16; *p* < 0.0001), with *dashed lines* denoting the 2SD confidence interval. *B*, the confirmed inhibitors of neuronal tau inclusions and multimers were grouped by target class based on the vendor-supplied annotation. Note that compounds could belong to more than one category.

To further verify the inhibition of tau inclusions in primary neurons, the validated non-toxic inhibitors were examined in a previously described orthogonal biochemical assay that measures multimeric tau species by ELISA (27). In this assay, AD-tau was again used to seed aggregation of tau pathology in rat cortical neuron cultures in the absence or presence of compounds as described for the primary screen, except that 96-well plates were used with compounds tested in duplicate. Culture lysates were then assessed in an ELISA assay in which the rodent tau-specific antibody mTau8 (see Experimental procedures and (27)) was used as both the capture and the detection antibody. Association of monomeric tau with immobilized capture antibody results in epitope shielding and the inability of the identical detection antibody to bind the tau, whereas multimeric tau species, including fibrils, can be detected because additional tau binding sites are available after association of tau with the capture antibody. All analyzed neuronal homogenates were within the linear range of the assay. Cytotoxicity of compounds was assessed by the determination of total protein levels in the cell lysate measured by a BCA assay. A compound hit threshold was established as ≥30% mean reduction in tau multimers relative to vehicle-treated neurons without evidence of appreciable (<25%) lowering of homogenate protein level. Nontoxic compounds that failed the mean activity criteria but showed ≥30% inhibition in one of the duplicate test wells were retested in triplicate and considered hits if ≥30% mean inhibition of tau multimers with <25% toxicity was observed. A total of 173 compounds were ultimately found to cause ≥30% reduction in tau multimers without evidence of appreciable toxicity (Fig. 2 and Table S1). A plot of the percent inhibition for each of the 271 hit compounds from the neuronal immunofluorescence assay *versus* inhibition in the tau multimer ELISA (Fig. 3*A*) reveals a number of compounds that failed to meet the ≥30% inhibitory threshold value in the latter assay, perhaps because many of these compounds act by mechanisms that reduce insoluble tau aggregates but not oligomeric tau species.

The 173 compounds that caused inhibition of both insoluble neuronal tau inclusions and tau multimers (Table S1) were grouped by their annotated biological targets and pathways, as summarized in Figure 3*B*. The results suggest that these compounds reduced tau pathology by altering a variety of cellular processes, including autophagy, neuronal signaling, metabolism, and multiple signal transduction pathways. Similarly, the annotation indicated that the inhibitors were directed to several target classes, including kinases, GPCRs, and ion channels. Finally, somewhat unexpected target classes were among those associated with the confirmed hits, including anti-infective agents. In addition to the list of confirmed inhibitors of tau neuronal pathology, *post hoc* examination of the primary screening data also revealed compounds that appeared to increase insoluble neuronal tau (*e.g.*, showed negative inhibition). The methodology utilized to quantify the integrated T49 immunofluorescence signal in the primary neuronal screen was optimized to identify inhibitors, which may have decreased the sensitivity for detecting compounds that elevated the T49 signal. Thus, the T49-stained immunofluorescence images of suspected enhancers were individually inspected to confirm evidence of increased tau aggregation, resulting in a small list of putative tau aggregate enhancers. These compounds were then examined for evidence of neurotoxicity through analysis of NeuN counts and MAP2 area, applying the toxicity thresholds described above. This resulted in a list of 15 potential non-toxic enhancers of neuronal tau pathology that were subsequently assessed in the mTau8 ELISA for evidence of increased neuronal tau multimeric species. A total of eight of the 15 compounds caused ≥30% increase in neuronal tau multimers relative to vehicle-treated neurons (Table S2). Interestingly, three of these enhancers of tau pathology are β-adrenergic receptor antagonists (bevantolol, betaxolol, and penbutolol).

### Concentration-response analyses of selected confirmed modulators of neuronal tau inclusions

The totality of tau inclusion inhibitors and enhancers was prioritized to select a set of compounds for repurchase in amounts to support full concentration-response testing in the primary neuronal assay. Priority was given to compounds that caused >40% inhibition of tau inclusions, those directed to druggable classes such as receptors or enzymes, and those for which multiple inhibitors were identified to the same target or pathway. Conversely, antibiotics and compounds known to act promiscuously, such as polyphenols, were not selected. A total of 63 compounds were re-ordered and underwent concentration-response testing in the primary screening assay and parallel assessments of neurotoxicity (55 inhibitors and 8 putative enhancers; Table 1). The majority of compounds showed evidence of a concentration-dependent change in neuronal tau inclusions with activity that was separated from measures of toxicity by approximately one log unit or more, with a total of 19 compounds showing “type 1” curves with definable lower and upper asymptotes and 30 compounds showing “type 2” curves with evidence of concentration-dependent activity without a clearly defined upper and/or lower boundary (Table 1 and Fig. S3). A total of 11 of the repurchased compounds showed evidence of activity that was not well separated from measures of toxicity, and three of the reordered compounds did not cause ≥30% inhibition although they were not toxic. Of note, the selected inhibitors of neuronal tau aggregates showed a high percentage of compounds (46 of 55) with evidence of concentration-dependent inhibition of tau inclusions that were separated from measures of toxicity, whereas only three of eight of the putative enhancers of tau pathology showed non-toxic activity (Table 1 and Fig. S3).

**Table 1:** Compounds repurchased for concentration-response testing

Compound type	Cat. No.	Product Name	Target	Pathway	Concentration-response profile
I	HY-10204	OSI-930	Apoptosis; c-Fms; c-Kit; VEGFR	Apoptosis; Protein Tyrosine Kinase/RTK	T
I	HY-10295	SB 202190	Apoptosis; Autophagy; p38 MAPK	Apoptosis; Autophagy; MAPK/ERK Pathway	2
I	HY-10342	Enzastaurin	Apoptosis; Autophagy; PKC	Apoptosis; Autophagy; Epigenetics; TGF-beta/Smad	1
I	HY-10435A	SKF-82958 (hydrobromide)	Dopamine Receptor	GPCR/G Protein; Neuronal Signaling	2
I	HY-10472	LY2811376	Beta-secretase	Neuronal Signaling	1
I	HY-107854	N-Acetyl-5-hydroxytryptamine	Endogenous Metabolite; Trk Receptor	Metabolic Enzyme/Protease; Neuronal Signaling; Protein Tyrosine Kinase/RTK	2
I	HY-11010	AS601245	JNK	MAPK/ERK Pathway	1
I	HY-112149	ZL0420	Epigenetic Reader Domain	Epigenetics	2
I	HY-112613	UCB9608	PI4K	PI3K/Akt/mTOR	1
I	HY-112667	CU-CPT-9a	Toll-like Receptor (TLR)	Immunology/Inflammation	2
I	HY-114269	(−)-(S)-B-973B	nAChR	Membrane Transporter/Ion Channel; Neuronal Signaling	1
E	HY-116790A	(+)-Penbutolol	Adrenergic Receptor	GPCR/G Protein; Neuronal Signaling	T
I	HY-117626	LP-935509	Others	Others	2
I	HY-12013	PD153035 (Hydrochloride)	EGFR	JAK/STAT Signaling; Protein Tyrosine Kinase/RTK	T
I	HY-120934	C25-140	E1/E2/E3 Enzyme; TNF Receptor	Apoptosis; Metabolic Enzyme/Protease	2
E	HY-121186	Bevantolol (hydrochloride)	Adrenergic Receptor; Calcium Channel	GPCR/G Protein; Membrane Transporter/Ion Channel; Neuronal Signaling	2
I	HY-122856	AZ12601011	TGF-β Receptor	TGF-beta/Smad	2
I	HY-123999	CD38 inhibitor 1	Others	Others	1
I	HY-12409	PFI-3	Epigenetic Reader Domain	Epigenetics	2
I	HY-12528	DBPR108	Dipeptidyl Peptidase	Metabolic Enzyme/Protease	1
E	HY-125837A	MS31 (trihydrochloride)	Epigenetic Reader Domain	Epigenetics	T
I	HY-126290	Ropsacitinib	JAK	Epigenetics; JAK/STAT Signaling; Stem Cell/Wnt	2
I	HY-12787	L-779450	Autophagy; Raf	Autophagy; MAPK/ERK Pathway	2
I	HY-12949	ML204	TRP Channel	Membrane Transporter/Ion Channel; Neuronal Signaling	2
I	HY-13288	Org 27,569	Cannabinoid Receptor	GPCR/G Protein; Neuronal Signaling	2
I	HY-13519	TRAM-34	Potassium Channel	Membrane Transporter/Ion Channel	2
I	HY-14581	Palomid 529	Apoptosis; mTOR	Apoptosis; PI3K/Akt/mTOR	1
I	HY-15124	(S)-(−)-Bay-K-8644	Calcium Channel	Membrane Transporter/Ion Channel; Neuronal Signaling	1
E	HY-15227	EPZ004777	Apoptosis; Histone Methyltransferase	Apoptosis; Epigenetics	T
I	HY-15427	GDC-0834	Btk	Protein Tyrosine Kinase/RTK	2
I	HY-15427B	GDC-0834 (S-enantiomer)	Btk	Protein Tyrosine Kinase/RTK	2
I	HY-15438	SB 415286	Apoptosis; GSK-3	Apoptosis; PI3K/Akt/mTOR; Stem Cell/Wnt	2
I	HY-15456	NVP-BVU972	c-Met/HGFR	Protein Tyrosine Kinase/RTK	2
I	HY-15681	Senexin A	CDK	Cell Cycle/DNA Damage	2
I	HY-16425	RG2833	HDAC	Cell Cycle/DNA Damage; Epigenetics	2
I	HY-18700	BRD73954	HDAC	Cell Cycle/DNA Damage; Epigenetics	2
I	HY-18938	Selonsertib	Apoptosis; MAP3K	Apoptosis; MAPK/ERK Pathway	1
I	HY-18976	UF010	HDAC	Cell Cycle/DNA Damage; Epigenetics	1
I	HY-19562	PF-06260933	MAP4K	MAPK/ERK Pathway	1
E	HY-19756	OTX008	Galectin	Immunology/Inflammation	2
I	HY-19834	Fenebrutinib	Btk	Protein Tyrosine Kinase/RTK	1
I	HY-19836	Zimlovisertib	IRAK	Immunology/Inflammation	T
I	HY-50858	Ruxolitinib (phosphate)	Autophagy; JAK; Mitophagy	Autophagy; Epigenetics; JAK/STAT Signaling; Stem Cell/Wnt	T
I	HY-50865	PDE-9 inhibitor	Phosphodiesterase (PDE)	Metabolic Enzyme/Protease	IA
I	HY-59090	1-Azakenpaullone	GSK-3	PI3K/Akt/mTOR; Stem Cell/Wnt	1
I	HY-70050C	Alosetron (Hydrochloride)	5-HT Receptor	GPCR/G Protein; Neuronal Signaling	1
I	HY-76772	Cevimeline (hydrochloride hemihydrate)	mAChR	GPCR/G Protein; Neuronal Signaling	2
I	HY-79511	FAAH-IN-2	Autophagy; FAAH	Autophagy; Metabolic Enzyme/Protease; Neuronal Signaling	1
I	HY-100482	CPI-637	Epigenetic Reader Domain; Histone Acetyltransferase	Epigenetics	2
I	HY-100726	GNE-272	Epigenetic Reader Domain; Histone Acetyltransferase	Epigenetics	1
I	HY-100740C	(1α,1′S,4β)-Lanabecestat	Beta-secretase	Neuronal Signaling	2
I	HY-101027	GSK 4027	Epigenetic Reader Domain; Histone Acetyltransferase	Epigenetics	IA
I	HY-101474A	Zanubrutinib	Btk	Protein Tyrosine Kinase/RTK	T
I	HY-101494	Temuterkib	ERK	MAPK/ERK Pathway; Stem Cell/Wnt	2
I	HY-101736	AMG9810	TRP Channel	Membrane Transporter/Ion Channel; Neuronal Signaling	T
I	HY-101855	Anle138b	Amyloid-β	Neuronal Signaling	2
I	HY-B0371 B	(R)-Terazosin	Adrenergic Receptor	GPCR/G Protein; Neuronal Signaling	1
I	HY-B0371D	(S)-Terazosin	Adrenergic Receptor	GPCR/G Protein; Neuronal Signaling	2
E	HY-B0381	Betaxolol	Adrenergic Receptor	GPCR/G Protein; Neuronal Signaling	2
I	HY-B0553	Methazolamide	Carbonic Anhydrase	Metabolic Enzyme/Protease	IA
I	HY-B0588	Brinzolamide	Carbonic Anhydrase	Metabolic Enzyme/Protease	1
E	HY-B0949	Protriptyline (hydrochloride)	AChE	Neuronal Signaling	T
E	HY-B1213	Trimipramine (maleate)	5-HT Receptor; Bacterial	Anti-infection; GPCR/G Protein; Neuronal Signaling	T

Compounds are identified as inhibitors (I) or enhancers (E) of neuronal tau pathology. The resulting concentration-response curve profile obtained for each compound is indicated, with 1 = *discernible upper and lower asymptotes*; 2 = *concentration-dependent inhibition without clear upper and/or lower asymptotes*; T = toxic; IA = inactive.

Among the compounds showing concentration-dependent modulation of neuronal tau inclusions were two or more directed to BACE1, Bruton’s tyrosine kinase (BTK), adrenergic receptors, and HDACs. Similarly, there were two or more compounds with concentration-dependent activity with reported mechanisms more broadly ascribed to pathways related to apoptosis, epigenetics, the cell cycle, or the MAPK/ERK pathway. As the tau inclusion inhibitors tested for concentration-dependent activity represent only ∼1/3 of the total number of confirmed non-toxic inhibitors (Table S1), it is likely that many of the additional confirmed hits would also cause concentration-dependent inhibition of tau inclusions. Further confirmation and mechanistic elucidation of the various drug targets and cellular pathways listed in Tables 1 and S1 will require additional studies beyond those described here, but this screening effort followed by confirmation testing, neurotoxicity assessment, and orthogonal analysis has yielded a substantial number of modulators of neuronal tau inclusions that merit further characterization in future studies.

### Compound effect on neuronal tau expression

**Figure 4:**
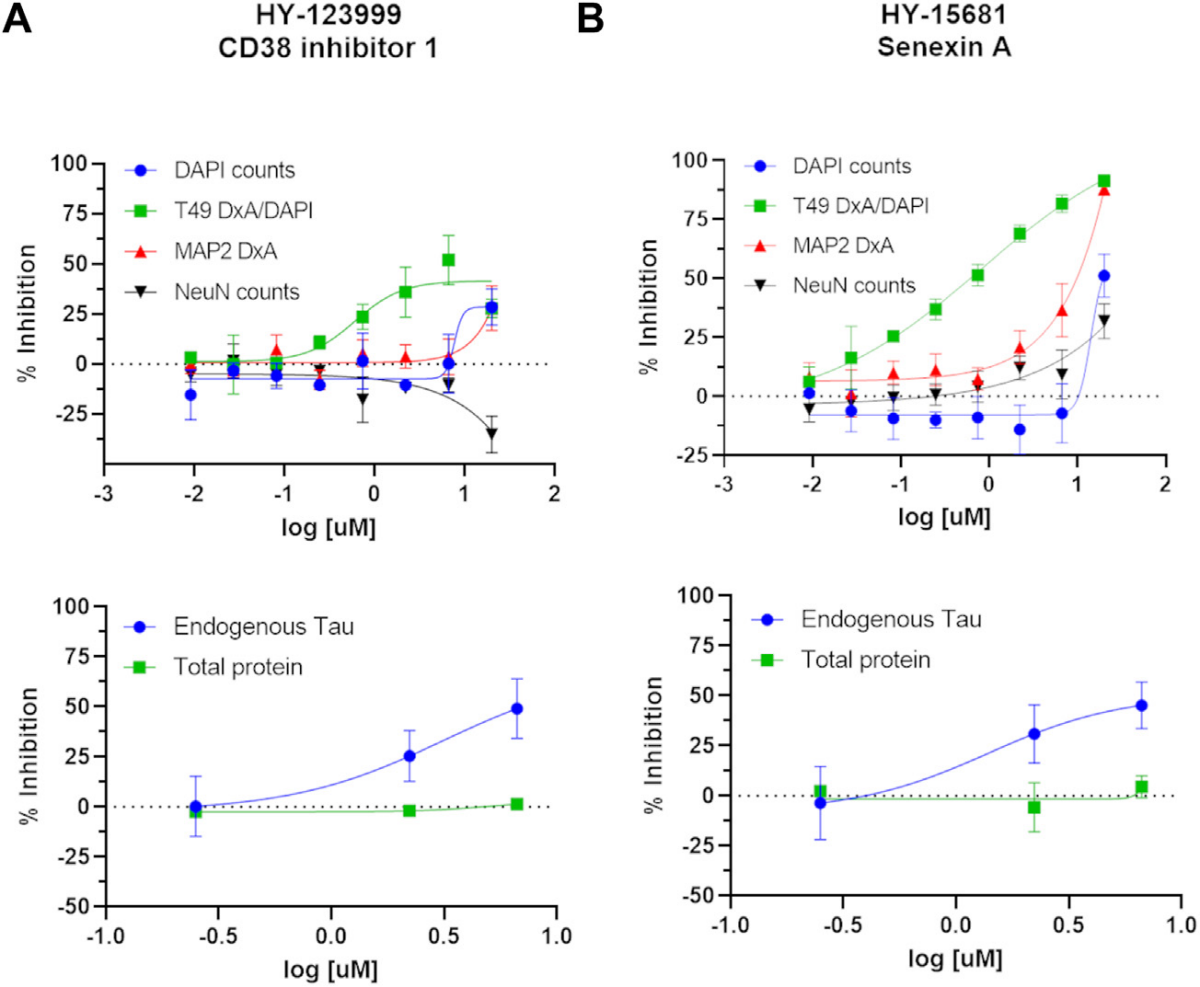
**Compounds that showed evidence of reducing total neuronal tau levels.***A*, HY-123999 and (*B*) HY-15681, with a comparison of the concentration-dependent inhibition of neuronal tau inclusions with associated toxicity measures (*top*) and the inhibition of soluble neuronal tau (*bottom*). In panels (*A*) and (*B*), the inhibition of tau pathology is denoted by the *green symbols* (integrated T49 staining normalized to total DAPI counts), whereas toxicity measures include the total DAPI counts (*blue*), integrated MAP2 signal (*red*) to assess dendritic processes, and total NeuN counts (*black*) to measure neuronal cell counts.

Compounds that alter neuronal tau aggregate formation could act by one of several mechanisms, including modulation of (1) exogenous AD-tau internalization; (2) intracellular trafficking of AD-tau after internalization; (3) tau fibril elongation; (4) tau fibril degradation; or (5) tau protein expression at the transcriptional or post-transcriptional levels. Although the full elucidation of compound mechanisms is beyond the scope of this study, we did examine whether compounds that caused a concentration-dependent decrease in tau inclusion formation also caused a reduction in neuronal tau protein levels (*i.e.*, point 5 above). A set of 30 inhibitory compounds with concentration-response profiles in which the second and third highest tested doses (*i.e.*, 6.7 and 2.2 μM) were relatively well separated from markers of neurotoxicity were tested at 0.24, 2.2, and 6.7 μM in primary rat cortical cultures plated in 96-well plates, with compound addition at DIV7 in the absence of AD-tau seeding. After an additional 14 days in culture (DIV21), cellular lysates were prepared for quantification of total soluble mouse tau by ELISA and total protein content by BCA assay. The highest concentration employed in the concentration-response testing with the tau inclusion assay (20 μM) was not utilized in this soluble tau assay because it was often associated with signs of neurotoxicity, whereas this was less frequent at the penultimate dose of 6.7 μM (*e.g.*, see Fig. S3). Significant decreases in total cellular tau (normalized to total protein in the lysate sample) would reflect changes in tau that could occur through changes in tau gene expression (*e.g.*, transcriptional changes or altered mRNA stability) or in soluble tau protein turnover. The majority of tested compounds did not significantly reduce neuronal tau levels in a concentration-dependent manner (Fig. S4), and thus these compounds are likely to modulate neuronal tau inclusions by an alternative mechanism. There were a few examples (*e.g.*, HY-10472, HY-13288, and HY15438) where apparent inhibition of soluble neuronal tau was observed at the lowest tested concentration but not at the higher doses. Although it is unclear why this inverse response was observed, it seems unlikely that these compounds act to lower tau inclusions through a lowering of soluble tau. Only two of the tested compounds showed evidence of a concentration-dependent change in total tau levels in the neuronal cultures (Fig. 4). Of these, HY-123999 (CD38 inhibitor 1) caused a reduction of endogenous neuronal tau that generally mirrored the inhibition of tau inclusion formation, with maximal inhibition of ∼50% for both measures and a general loss of inhibitory activity below 1 μM concentration (Fig. 4*A*). In contrast, HY-15681 (Senexin A) showed a lesser maximal lowering of soluble neuronal tau (∼50%) than inhibition of tau inclusions (∼85%), and there appeared to be no reduction of endogenous tau levels at 0.24 μM compound concentration whereas appreciable inhibition of neuronal tau inclusions was still evident at this concentration (Fig. 4*B*). The differences in the inhibition profiles of Senexin A for soluble tau and tau inclusions may be due to the different culture formats used to assess endogenous neuronal tau (96-well plates) and neuronal tau inclusions (384-well plates). However, it is also possible that the primary mechanism of tau aggregate lowering by Senexin A is unrelated to the apparent partial lowering of endogenous neuronal tau.

### Effect of BACE1 and γ-secretase inhibitors on neuronal tau inclusions

**Figure 5:**
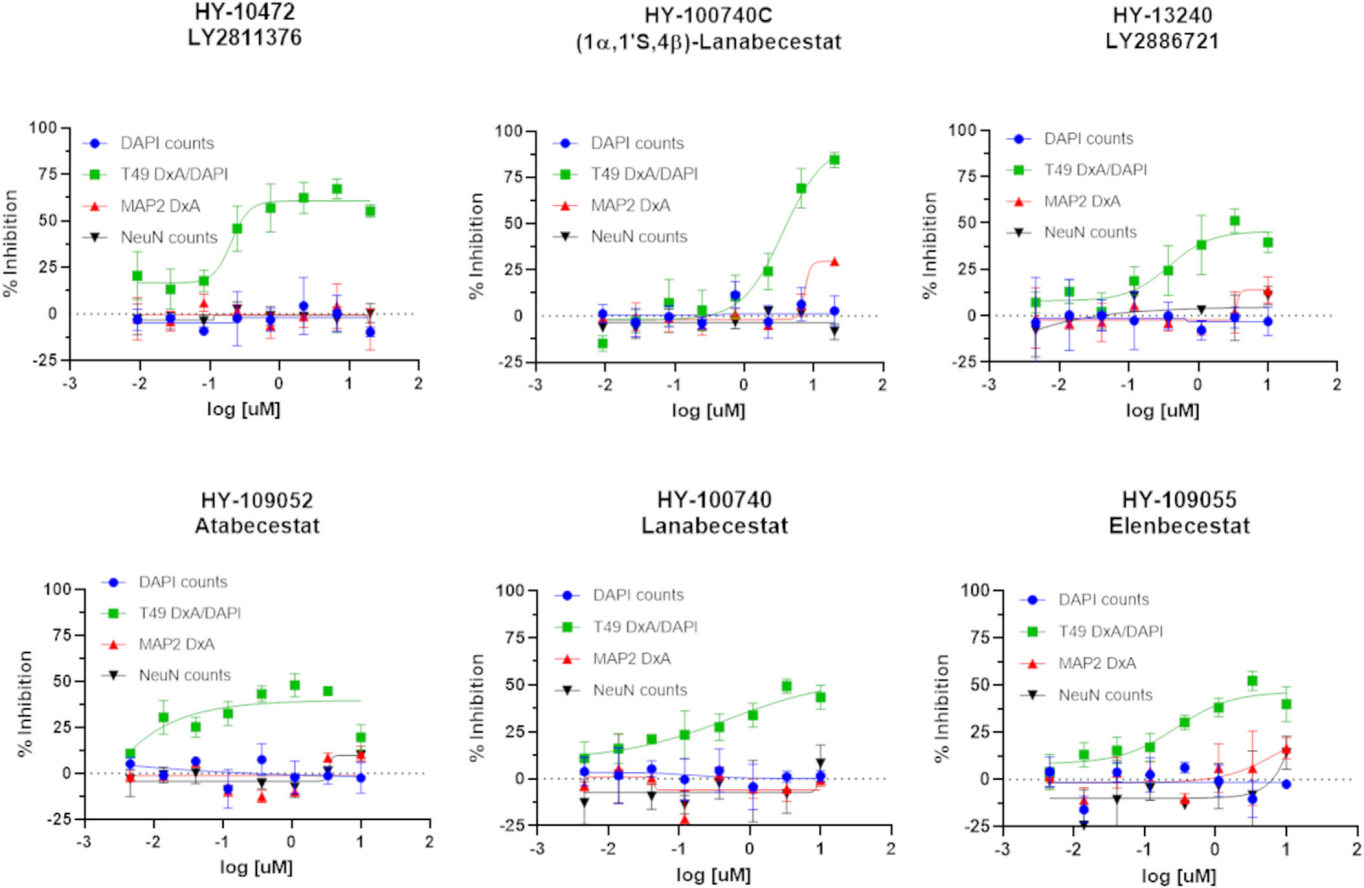
**BACE1 inhibitors that caused concentration-dependent reduction of neuronal tau inclusions.** The inhibition curves for T49-positive tau inclusions are shown in *green*. Measures of toxicity include DAPI-positive nuclei (*blue*), NeuN-positive neurons (*black*) and MAP2-positive dendritic process density (*red*).

Among the compounds causing inhibition of neuronal tau inclusions and multimers are the BACE1 inhibitors LY2811376, (1α,1′S,4β)-Lanabecestat and LY2886721 (Table S1 and Fig. S3). Compounds of this type have been developed as potential AD therapeutics due to the role of BACE1 in APP cleavage and generation of the Aβ peptides found in senile plaques (37). As it is known that Aβ senile plaque pathology can hasten the formation of tau pathology in mouse models of AD (38, 39), BACE1 inhibitors are predicted to reduce plaque-mediated enhancement of tau pathology. However, to our knowledge, BACE1 activity or Aβ peptides have not been implicated in the regulation of insoluble tau inclusions in neuronal culture systems like that employed here, although exogenously applied Aβ has been reported to affect neuronal tau phosphorylation (40, 41, 42). There were additional BACE1 inhibitors within the MCE library that were not identified as confirmed hits, with some exceeding the neurotoxicity criteria at the tested 10 μM concentration. In addition, the BACE1 inhibitors atabecestat and elenbecestat failed to meet the >3SD inhibition hit threshold in the primary screen, but each had one or more wells in triplicate testing with >40% inhibition of tau pathology. Finally, the BACE1 inhibitor lanabecestat showed evidence of reducing tau inclusions but fell just below the ≥30% inhibition cutoff in the primary screen (28 ± 3%). We thus examined the concentration-response profiles of these additional BACE1 inhibitors in the neuronal tau inclusion assay. The results revealed that these BACE1 inhibitors, as well as the previously identified confirmed hits LY2811376, (1α,1′S,4β)-Lanabecestat and LY2886721, showed evidence of a concentration-dependent inhibition of neuronal tau inclusions that reached a maximal reduction of ∼40 to 70% (Fig. 5). Interestingly, additional studies showed that the concentration-response profiles of the BACE1 inhibitors were similar when the compounds were added 1 day after seeding of the neurons with AD-tau (not shown). This suggests that the BACE1 inhibitor reduction of neuronal tau pathology was not *via* an effect on AD-tau internalization or initial seeding.

**Figure 6:**
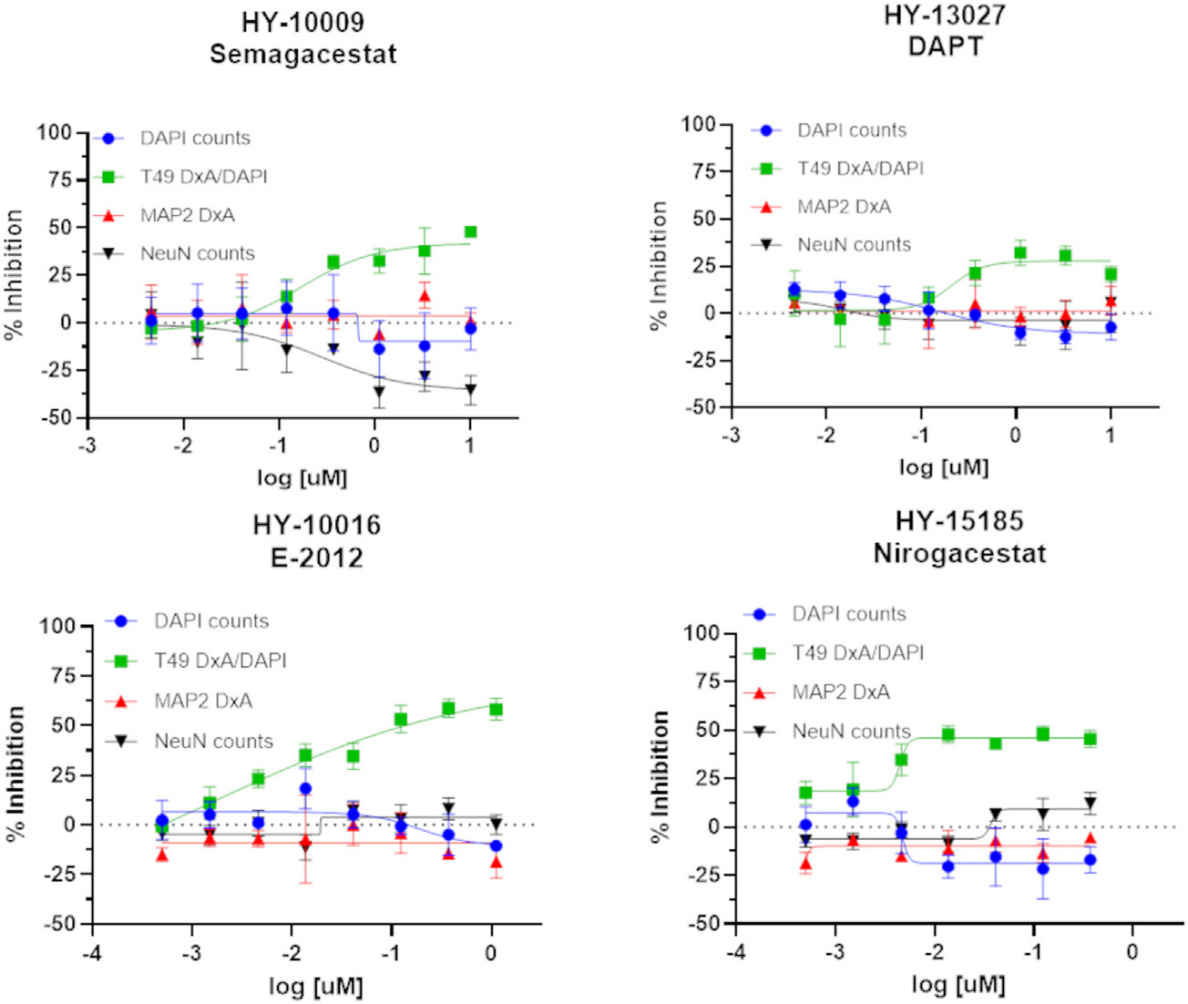
**Concentration-dependent inhibition of neuronal tau inclusions by γ-secretase inhibitors.** Inhibitors that caused concentration-dependent reduction of neuronal tau inclusions are depicted, with inhibition curves for T49-positive tau inclusions shown in *green*. Measures of toxicity include DAPI-positive nuclei (*blue*), NeuN-positive neurons (*black*) and dendritic density (*red*).

**Figure 7:**
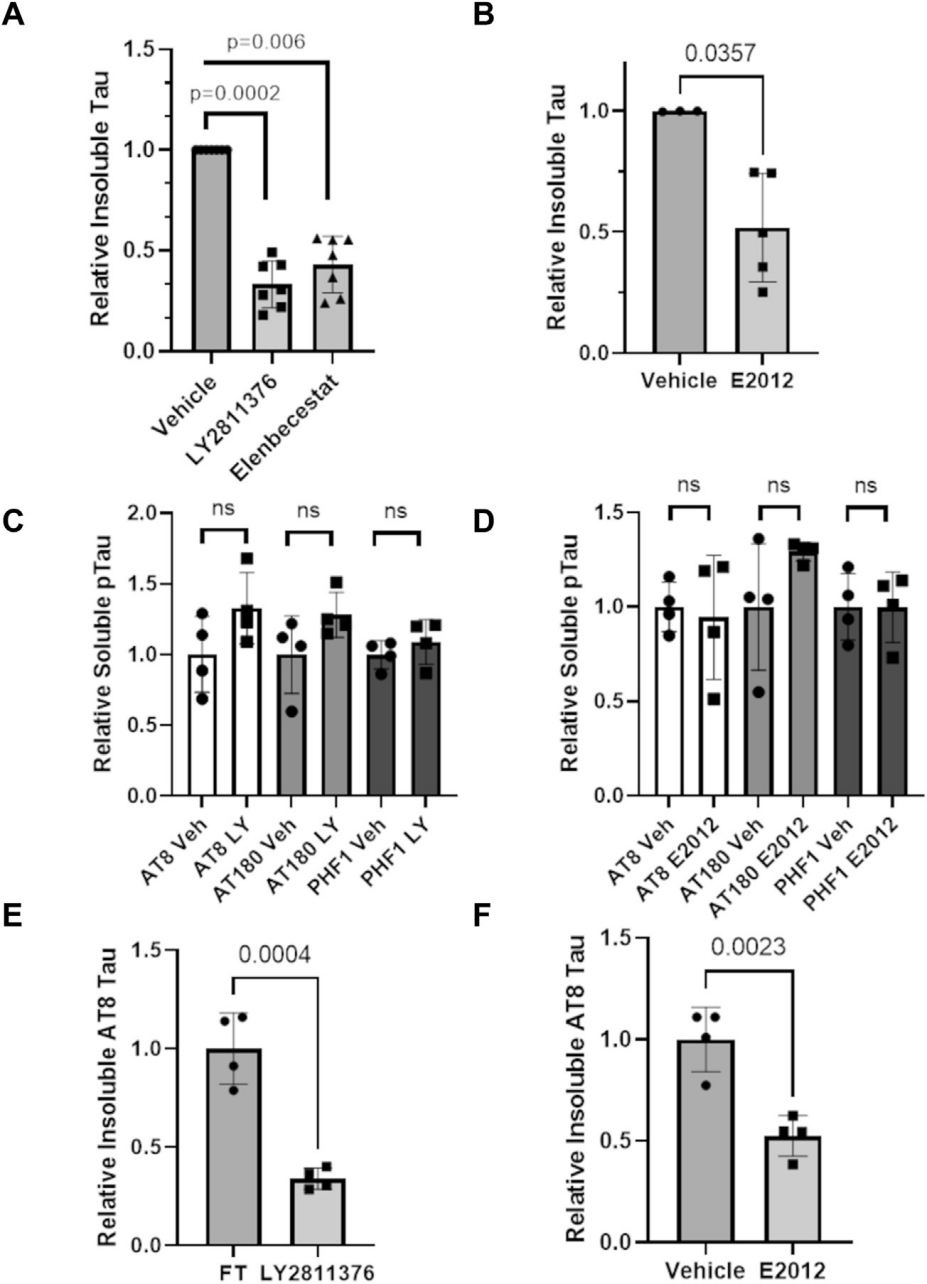
**BACE1 and γ-secretase inhibitors reduce total insoluble tau and phospho-tau, but not soluble phospho-tau.***A*, the BACE1 inhibitors LY2811376 and Elenbecestat (3.3 μM) and (*B*) the γ-secretase inhibitor E20212 (1 μM) caused significant reductions of total insoluble tau as assessed by immunoblot using the T49 antibody (see also Fig. S5).) Neither (*C*) the BACE1 inhibitor LY2811376 (LY) or (*D*) the γ-secretase inhibitor E20212 reduced AT8-, AT180-, or PHF1-positive soluble phospho-tau. In contrast, insoluble AT8-positive phospho-tau was diminished by treatment with (*E*) LY2811376 and (*F*) E2012. A non-parametric Kruskal-Wallis analysis was used in panel (*A*) and a non-parametric Mann-Whitney test was used in panel (*B*), as compound-treated values were normalized to the corresponding vehicle value on individual blots used for the analyses. Unpaired two-tailed t-tests were used for panels (*C*–*F*). Each symbol represents independent samples, with error bars denoting standard deviation.

The observation that multiple BACE1 inhibitors reduced tau pathology suggested that they acted *via* an on-target mechanism and that a BACE1 cleavage product promoted tau inclusion formation. If the reduction in tau inclusions upon BACE1 inhibitor treatment occurred through a reduction of APP cleavage and perhaps Aβ generation, it would be expected that inhibitors/modulators of γ-secretase activity would also reduce neuronal tau aggregates, as this enzyme complex releases Aβ from the BACE1-generated APP fragment. The screened library contains several γ-secretase inhibitors/modulators which failed to meet the inhibition threshold in the primary screen or caused toxicity at 10 μM. However, semagacestat and crenigacestat were found to have effectively inhibited neuronal tau inclusions both in the primary screen and upon confirmation activity testing without evidence of neurotoxicity, although these compounds failed to make the confirmed hit list because they did not meet the activity threshold in the orthogonal tau oligomer ELISA. In addition, the reported γ-secretase inhibitor, DAPT, showed inhibition of tau pathology in the primary screen without evidence of toxicity, although this compound failed to meet the activity threshold upon confirmation testing. To determine whether assay variability may have led to lower than expected activity of these compounds upon confirmation or orthogonal testing, we retested them in triplicate at 10 μM in the mTau8 multimer ELISA. Notably, all three of these reported γ-secretase inhibitors caused a significant reduction of tau multimers without evidence of meaningful toxicity upon retesting (Table S3). We subsequently examined the concentration-response profiles of these compounds, as well as additional γ-secretase inhibitors/modulators within the library that showed toxicity at the 10 μM screening concentration to determine whether such compounds might show evidence of non-toxic tau inclusion inhibition at lower concentrations. Four of the tested γ-secretase inhibitors/modulators (semagacestat, DAPT, E−2012, and nirogacestat) consistently showed evidence of a concentration-dependent reduction of tau inclusions that was separated from concentrations that elicited neurotoxicity (Fig. 6). The magnitude of inhibition of tau pathology (∼30–50%) with the γ-secretase inhibitors/modulators was generally somewhat less than that observed with the BACE1 inhibitors (40–70%). To further confirm that BACE1 and γ-secretase inhibitors were decreasing insoluble tau, additional studies were conducted in which the amount of rat tau within soluble and insoluble fractions from AD-tau-treated neuronal culture homogenates was assessed by immunoblotting. As summarized in Figure 7*A*, the two tested BACE1 inhibitors, LY2811376 and elenbecestat (at 3.3 μM), caused significant reductions in total insoluble neuronal tau, whereas they did not decrease the amount of soluble tau (Fig. S5*A*). Similarly, the tested γ-secretase inhibitor E2012 (1 μM) also caused a significant reduction of insoluble tau without decreasing soluble tau levels (Figs. 7*B* and S5*B*).

The observation that inhibitors of BACE1 and γ-secretase reduced neuronal tau pathology suggested a possible mechanism of reduced APP cleavage. In an attempt to determine whether an APP cleavage product regulated neuronal tau pathology, multiple studies were conducted with siRNA directed to rat APP. Treatment of the AD-tau seeded neuronal cultures with a pool of four rat APP siRNA at DIV7 led to dose-dependent decrease of APP protein in the cultures at DIV21, with 1 μM pooled APP siRNA causing ∼80% reduction of APP (not shown). However, studies examining the effect of APP knockdown on neuronal tau pathology were ultimately inconclusive. Whereas results from the 384-well immunocytochemical assay used in screening revealed evidence of a reduction of tau pathology that was greater upon the addition of APP siRNA than control siRNA, subsequent immunoblot analyses did not show clear differentiation in amounts of insoluble tau between APP and control siRNA treated neuron cultures. This was due in part to the control siRNA causing a variable reduction of neuronal tau inclusions. Thus, future experimentation using alternative approaches will be required to conclusively determine whether the BACE1 and γ-secretase inhibition of neuronal tau inclusions is mediated through reduced APP processing.

To further explore how BACE1 and γ-secretase inhibition might reduce tau inclusions, we examined the effect of inhibiting these enzymes on tau phosphorylation. Prior studies have shown that exogenously added oligomeric or fibrillar Aβ species can promote neuronal tau phosphorylation (40, 41), and hyperphosphorylation of tau is thought to contribute to the formation of pathology due to increased tau disengagement from microtubules and a resulting increase in cytosolic phosphorylated tau that can assemble into tau fibrils (43). An examination of soluble tau phosphorylation in AD-tau-treated neuron cultures receiving the BACE1 inhibitor LY2811376 or the γ-secretase inhibitor E2012 revealed that phosphorylation at the S202/T205 (AT8 antibody), T231 (AT180) and S396/S404 (PHF1) tau residues were not reduced by inhibitor treatment (Figs. 7, *C* and *D* and S6). Although the assessment of phosphorylated tau species in the insoluble neuronal fraction is somewhat complicated by the presence of residual exogenous phosphorylated AD-tau in the cultures at DIV21, particularly for the AT180 and PHF1 epitopes, we observed that insoluble AT8-positive tau (Fig. S7) was reduced upon treatment with the BACE1 inhibitor (Fig. 7*E*) or γ-secretase inhibitor (Fig. 7*F*). This result is in alignment with the reductions in total insoluble tau caused by these inhibitors (Fig. 7, *A* and *B*). Thus, while BACE1 and γ-secretase inhibitors reduce insoluble neuronal tau, they do not act by reducing soluble tau phosphorylation.

## Discussion

The screening of an ∼8700 compound library with mechanistic annotation at 10 μM concentration in a primary rat cortical neuron assay of tau inclusion formation, followed by subsequent neurotoxicity and orthogonal activity testing, led to the identification of 173 inhibitors that reduced tau aggregates and multimers by ≥30% without appreciable neurotoxicity. In addition, a small number of compounds were identified upon *post hoc* analysis that appeared to enhance neuronal tau inclusions. A selection of 55 confirmed inhibitors and eight putative enhancers of tau pathology were repurchased in larger quantities and examined for their ability to cause a concentration-dependent alteration of neuronal tau inclusions. Gratifyingly, 46 of the 55 tested inhibitors caused a concentration-dependent lowering of tau inclusions with activity separated from measures of neurotoxicity. In addition, three enhancers of tau pathology were found to promote more tau inclusions with increasing concentration.

The compound annotation provided by the library vendor indicates that the identified inhibitors and enhancers of tau pathology are directed to a number of different cellular processes. These include autophagy, specific signal transduction pathways, and metabolic pathways. Similarly, the active compounds were directed to several protein target classes, including kinases, GPCRs, and ion channels. Finally, active compounds were identified that belong to unexpected classes, including anti-infective agents. The majority of compounds that caused a concentration-dependent inhibition or enhancement of neuronal tau inclusions had apparent EC_50_ values (concentration eliciting 50% of maximal activity) of >1 μM, although there were examples with sub-μM EC_50_ values. As many of the active tau inclusion inhibitors/enhancers directed to receptors or enzymes have reported sub-μM target affinity, the lower potency in the neuronal tau inclusion model might suggest that these compounds are acting *via* off-target interactions. However, it is also possible that the EC_50_ values observed in the neuronal assay underestimate the true target binding affinity, as the effect on tau inclusions is measured 14 days after a single compound addition. Thus, the effective compound concentration in the culture medium may be diminished due to metabolism, chemical changes (*e.g.*, redox events), or non-specific binding, resulting in a rightward shift in the concentration-response profile.

The majority of compounds that caused a concentration-dependent decrease in neuronal tau pathology were tested to assess whether any might act by lowering neuronal soluble tau levels. Most of the tested compounds did not lead to a significant lowering of total neuronal tau, with only two compounds showing evidence of a concentration-dependent reduction of soluble neuronal tau in the absence of toxicity. These data indicate that the majority of neuronal tau inclusion inhibitors act by alternative mechanisms, although compounds that do lower neuronal tau levels could still be interesting candidates for further study, as reducing neuronal tau expression through the use of ASOs has been suggested as a therapeutic strategy for AD and other tauopathies (44).

In most instances, only a fraction of the compounds directed to a given target within the library were identified as non-toxic inhibitors of neuronal tau inclusions and multimers. For example, although multiple examples of BACE1 and BTK inhibitors were found to reduce neuronal tau aggregates and multimers, additional BACE1 and BTK inhibitors within the compound library did not meet the assay hit criteria. Many compounds that were directed to the same target as a confirmed hit compound were eliminated due to evidence of toxicity. Moreover, in other instances, compounds showed measurable activity that did not quite meet one of the assay activity criteria. In subsequent follow-up studies, additional BACE1 inhibitors and γ-secretase inhibitors that were either toxic in the initial screen or failed activity criteria were found to cause a concentration-dependent inhibition of neuronal tau pathology. Indeed, the additional investigation of BACE1 and γ-secretase inhibitors that were not initially identified as confirmed hits reveal some of the limitations of HTS campaigns. For example, compounds that were found to be toxic at the 10 μM screening concentration may display non-toxic inhibition of neuronal tau inclusions at lower concentrations. Moreover, potentially active compounds could be missed due to the hit criteria being set at a level that excluded weakly active compounds, or because an outlier value in one of the triplicate wells lowered the mean activity below the hit threshold. Thus, although screening compounds in triplicate provides greater information than would be achieved in typical screens where compounds are examined in a single assay well, false negative results can occur. In this regard, we have previously conducted a biochemical screen using a quantitative HTS (qHTS) method in which every compound was assessed at multiple concentrations (45). Although this qHTS method provides a more thorough analysis of each compound that reduces the likelihood of inappropriate exclusion of potential hits, this more labor-intensive approach is not generally practical for complicated cellular assays such as the neuronal tau inclusion model used here.

A prior screen of the Prestwick library of mostly approved drugs in the neuronal tau inclusion assay (27) resulted in the identification of four dopamine D2 receptor antagonists that caused a concentration-dependent inhibition of neuronal tau pathology, and surprisingly D2 antagonists were not among the confirmed non-toxic inhibitors of tau inclusions identified in the current screen. A search revealed that three of the four previously identified active D2 antagonists from the Prestwick screen (metoclopramide, alizapride, and azaperone) were within the library utilized in the current screen. An inspection of the primary screening data revealed that azaperone was excluded due to excessive toxicity, and metoclopramide failed to reach the 30% inhibition hit threshold because one of the triplicate wells failed to show appreciable inhibition, whereas the other two wells had >30% inhibition of neuronal tau aggregates. Similarly, alizapride failed to meet the 30% hit criteria although one of three wells showed 35% inhibition. It is unfortunate that well-to-well variability led to the exclusion of metoclopramide and alizapride from the current hit list, and although the utilization of triplicate wells in the screen likely decreased the number of false positive hits, as noted above false-negative results can still occur. These examples again reveal some of the limitations of HTS, and it is clear that in many instances additional analyses will be required for targets of potential interest, including reexamination of additional compounds directed to the same target that did not initially pass the activity or toxicity thresholds. In addition, target knockdown and/or overexpression studies can provide additional understanding of the active compound mechanism.

The more thorough analyses of BACE1 and γ-secretase inhibitors suggest that the proteolytic processing of a shared substrate, perhaps APP, leads to enhanced tau pathology. Unfortunately, studies with APP siRNA to reduce APP expression were inconclusive. Although appreciable and specific APP knockdown was observed in siRNA-treated neuron cultures, the effect on insoluble tau levels was inconsistent and complicated in part by instances of control siRNA causing appreciable non-specific reduction of tau pathology. Thus, additional future studies will be required to determine whether a BACE1-and γ-secretase-generated APP fragment, such as Aβ, can modulate tau pathology. Multiple studies in mouse models have demonstrated that Aβ plaques can exacerbate tau pathology (38, 39) and there are prior reports of exogenously added Aβ species promoting neuronal tau phosphorylation and/or misfolding (40, 41, 42, 46). Given this literature, it is interesting that we did not observe a decrease of phosphorylated tau species upon treatment of neurons with BACE1 or γ-secretase inhibitors. It is also possible that an alternative APP fragment modulates tau pathology. For example, the processing of APP by BACE1 and γ-secretase generates intracellular AICD fragments that have been reported to have biological activity, including transcriptional regulation (47). Finally, we cannot exclude the possibility that these enzymes act on another shared substrate or perhaps by independent mechanisms. In addition to future studies directed to further elucidation of BACE1 and γ-secretase modulation of neuronal tau pathology *in vitro*, it will also be important to determine whether inhibition of these enzymes leads to a reduction of tau pathology in mouse models of tauopathy. If the observations made in the neuronal culture system described here are replicated *in vivo*, then modulators of APP processing might hold promise for pure tauopathies, as well as AD. However, the well-documented safety issues observed with existing BACE1 (37) and γ-secretase (48) inhibitors would have to be overcome to allow future testing of such compounds in tauopathy patients.

In conclusion, a number of modulators of neuronal tau pathology have been identified through the screening of an annotated library of biologically active compounds in a rat cortical neuron model of seeded tau inclusion formation. The identification of compounds and related targets that affect neuronal tau pathology provides an important starting point for future studies that could lead to the identification of pathways and targets involved in the regulation of tau pathology.

## Experimental procedures

### Compound library preparation

The Bioactive Compound Library (MedChem Express, HY-L001) comprised of 8663 compounds was utilized for screening, with compounds supplied at 10 mM in dimethylsulfoxide (DMSO, Sigma-Aldrich), except for 126 compounds that were provided at 2 mM stock concentration in DMSO, 33 compounds provided at 3 mg/ml in DMSO, and nine compounds provided at 3 mg/ml in water. Daughter plates were prepared from the originally supplied compound plates, with compounds arrayed at 5 mM in DMSO with three replicates per 384-well plate in non-consecutive wells (*e.g.*, plate locations C5, C10, C15) with DMSO-only vehicle controls in columns, 3, 4, 21, and 22. Compounds that underwent concentration-response testing were plated in 384-well plates in triplicate at 10 or 20 μM in DMSO with 3-fold dilutions downward in DMSO to yield eight concentrations. Compound plates for both screening and concentration-response experiments were heat sealed with foil and stored at −20 °C and thawed at 22 °C at least 1 h prior to use, with a final 500-fold dilution after addition to neurons as described below. For analysis of confirmed non-toxic hit compounds in a tau multimer ELISA, compounds were picked from the daughter plates described above and plated in 96-well plates at 40 μM prior to addition to neurons as described below. All of the above conditions, with the exception of the nine compounds provided in aqueous solution, resulted in an identical dilution scheme of 1/500 from DMSO stock for a final DMSO concentration of 0.2% in the culture medium. A small subset of compounds that failed to reach the threshold hit definition of >30% mean inhibition of tau multimers where one of the duplicate values showed >30% inhibition of multimeric tau were retested in triplicate.

### AD-tau extraction

AD-tau was prepared using a previously described method in which enriched preparations of insoluble human tau with little contamination of Aβ or α-synuclein are prepared from AD brains (28). AD cases were selected based on high pathological tau burden and lack of co-morbid proteinopathies including α-synuclein and TDP-43 aggregates. Autopsied brain tissues were obtained after obtaining legal consent from the next of kin for tissue donation to the Center for Neurodegenerative Disease Brain Bank at the University of Pennsylvania (49). Frozen frontal cortex tissue was thawed and gray matter separated from white matter. Protease inhibitors were prepared to consist of 1 mg/ml pepstatin A (Sigma-Aldrich), 1 mg/ml leupeptin (Sigma-Aldrich), 1 mg/ml N-p-tosyl-L-phenylalanine chloromethylketone (TPCK, Sigma-Aldrich), 1 mg/ml N_α_-tosyl-L-lysine chloromethyl ketone hydrochloride (TLCK, Sigma-Aldrich), 1 mg/ml trypsin inhibitor (Sigma-Aldrich), and 100 mM ethylenediaminetetraacetic acid (EDTA, Sigma-Aldrich). Phosphatase inhibitors were prepared consisting of 200 mM imidazole (Sigma-Aldrich), 100 mM sodium fluoride (Sigma-Aldrich), and 100 mM sodium orthovanadate (Sigma-Aldrich). The gray matter was weighed and homogenized in 9x volume-to-weight of PHF buffer consisting of 10 mM Tris pH 7.4 (Fisher), 800 mM sodium chloride (Fisher), 1 mM EDTA (Sigma-Aldrich), 10% sucrose (Sigma-Aldrich), 0.1% N-Lauroylsarcosine sodium salt (sarkosyl, Sigma-Aldrich), 2 mM dithiothreitol (DTT, Fisher), 0.1 mM phenylmethylsulfonyl fluoride (PMSF, Sigma-Aldrich), 1:1000 diluted protease inhibitor and 1:100 diluted phosphatase inhibitor. Tissue was dissociated in a dounce homogenizer on ice. Homogenate was centrifuged at 10,000*g* for 10 min at 4 °C. The supernatant was collected and the sarkosyl concentration was increased to 1% by the addition of 25% sarkosyl solution to PHF buffer and stirred at 22 °C for 1 h. Homogenate was centrifuged at 150,000*g* for 75 min at 4 °C. The pellet was rinsed with phosphate-buffered saline pH 7.4 (PBS) and then resuspended in PBS and centrifuged at 250,000*g* for 30 min at 4 °C. The resulting final pellet was resuspended in 0. 1 ml PBS per gram of original gray matter with rotation at 4 °C overnight, then passed through a 27-gauge needle and sonicated with 30 pulses on power 2 with a probe sonicator (QSonica, XL-2000). These AD-tau enriched preparations were aliquoted and stored at −80 °C.

### Primary neuron culture

Tissue culture 10 cm dishes (Corning #353003), 12-well plates and 96-well plates (PerkinElmer #6005182) were treated with 10 ml, 1 ml/well or 150 μl/well, respectively, of 0.1 mg/ml Poly-D-lysine (PDL; Sigma-Aldrich) in 50 mM Borate buffer pH 8.5 (Fisher) at 22 °C overnight or 37 °C for 3 h. Plates were washed five times with 10 ml, 1 ml, or 250 μl, respectively, using cell culture application water (Lonza). 384-well plates were purchased pre-coated with PDL (Corning #356663). Rat cortical neuron preparations were either supplied by a core facility at the University of Pennsylvania or prepared in-house following protocols approved by the Institutional Animal Care and Use Committee. Rat (Sprague-Dawley, Charles Rivers) E18 embryos were dissected, and cortex tissue was digested in 0.25% Trypsin-EDTA solution (Gibco) for 20 min at 37 °C. Digestion was quenched with 10% fetal bovine serum (Sigma, F2442) in DMEM (Gibco), and cells were gently triturated and filtered through a cell strainer (Falcon). Cells were plated at a density of 5 × 10^6^ cells/10 cm dish with sterile filtered complete neuronal media containing Neurobasal medium (Gibco) supplemented with 2% B-27 (Gibco), 1% GlutaMAX (Gibco), and 1% Penicillin-Streptomycin (Gibco). Cells were plated at a density of 20,000 cells/well in 96-well plates, 90,000 cells/well in 12-well plates and 5000 cells/well in 384-well plates in complete neuronal media supplemented with 5% fetal bovine serum (FBS; Atlanta Biologicals). To avoid evaporative effects, cells are plated only in internal wells in plates, filling the outermost wells with sterile water. Cells were incubated at 37 °C with 5% CO_2_ in a humidified incubator (Thermo Fisher Scientific, HERAcell 150i). After 1 day *in vitro* (DIV1) media in 12-, 96- and 384-well plates was exchanged to complete neuronal media without FBS such that plates contained 1 ml/well, 100 μl/well and 50 μl/well, respectively.

### Compound treatment and AD-tau transduction

Conditioned media was prepared by collecting media from neuron cultures at DIV7 that were grown on 10-cm dishes, which was diluted with an equal volume of complete neuronal media and sterile-filtered. For neurons grown on 384-well plates, at DIV7 25 μl of media was removed from each well and 0.1 μl of 5 mM compound in DMSO was added for screening (or 500× the respective final concentration for concentration-response analyses) using a 384-well pin tool dispenser (0.2% final DMSO in all wells) and cells were incubated at 37 °C for 1 h. AD-tau was diluted to 40 μg of total tau per ml in PBS and sonicated in a water bath sonicator (Diagenode) for 20 cycles of 30 s on and 30 s off at 9 °C. The sonicated AD-tau was then diluted to 2.5 μg/ml in conditioned media and 25 μl/well was added to cells, providing 62.5 ng/well AD-tau and a final volume of 50 μl. The same pooled AD-tau preparation was used throughout the primary screening of the complete compound library. A second pooled AD-tau preparation that was prepared using the same protocol was used in some of the later studies, with the concentration of final AD-tau adjusted per well to achieve the same amount of pathology as the initial AD-tau preparation. Cells were incubated for 14 days, without exchanging media or adding additional compound, at 37 °C with 5% CO_2_ in a humidified incubator. Peripheral water wells were refilled if extensive evaporation was observed. For the 96-well plate tau multimer ELISA assays, the compounds were diluted from 5 mM DMSO stock solutions to 40 μM in complete neuronal media. Prior to the addition of compounds to DIV7 neurons grown in 96-well plates, 50 μl of 100 μl of total media was removed from each well, and 25 μl of 40 μM compound added (0.27% final DMSO) and incubated at 37 °C for 1 h. AD-tau was diluted in PBS, using twice the amount used for 384-well plates to scale for per-well volume differences, with sonication as described above followed by dilution in conditioned media and the addition of 25 μl/well for a final volume of 100 μl. For the 12-well plate immunoblot assays, compounds were diluted from 5 mM DMSO stock solutions to achieve a non-toxic active concentration. For BACE1 inhibitors, 13.2 μM compound was prepared in complete neuronal media. After the removal of 0.5 ml of media from a total of 1 ml media/well, 0.25 ml of the compound-containing medium was added. For γ-secretase inhibitors, 4 μM compound was prepared in complete neuronal media and added as described for the BACE1 inhibitors. Compounds were incubated at 37 °C for 1 h, followed by addition of 0.25 ml of AD-tau that was prepared as described above for 384-well assays using 20-fold more AD-tau to scale for differences in medium volume between the 12-well and 384-well plates. The final concentrations of BACE1 and γ-secretase inhibitor concentrations were 3.3 μM and 1 μM, respectively.

### Immunocytochemistry

Cells in 384-well plates were washed five times with PBS using an automated plate washer (Bio-Tek ELx405) with 25 μl of PBS remaining after the final wash. To extract soluble protein prior to staining of insoluble rat tau pathology, 25 μl of 2% hexadecyltriethylammonium bromide (HDTA, Sigma-Aldrich) at 20 °C was added *via* liquid handling robotics (PerkinElmer Evolution P3) and incubated at 22 °C for 10 min. For fixation, HDTA solution was removed from wells and 25 μl of 8% paraformaldehyde (PFA, Electron Microscopy Sciences) and 8% sucrose in PBS were added to cells and incubated at 22 °C for 20 min. Cells were washed five times with PBS and treated with a blocking buffer comprised of 3% bovine serum albumin (BSA, Sigma-Aldrich) and 3% FBS (Corning) in PBS at 22 °C for 1 h. Insoluble pathological rat tau was stained with T49 antibody (27), with ascites diluted 1:10,000 in blocking buffer at 22 °C for 1 h or overnight at 4 °C. Cells were washed five times with PBS and treated with goat anti-mouse 488 (Invitrogen) secondary antibody diluted to 0.2 mg/ml and 0.83 mg/ml DAPI (Invitrogen) in blocking buffer at 22 °C for 1 h.

For MAP2 and NeuN staining to assess neuronal health, cells were washed five times with PBS and then left with 25 μl of remaining PBS. Cells were fixed and permeabilized by addition of 25 μl of 8% PFA, 8% sucrose, and 0.25% Triton X-100 (Sigma-Aldrich) in PBS at 22 °C for 20 min. Cells were washed five times with PBS and treated with 25 μl of blocking buffer at 22 °C for 1 h. Cells were stained with MAP2 antibody (in house rabbit polyclonal serum for antibody 17028) diluted 1:2500 and NeuN (Millipore) diluted 1:1000 in blocking buffer and incubated at 22 °C for 1 h or overnight at 4 °C. Cells were washed five times with PBS and treated with goat anti-mouse 488 and goat anti-rabbit 594 (Invitrogen) secondary antibody, each diluted to 0.2 mg/ml, and 0.83 mg/ml DAPI in blocking buffer at 22 °C for 1 h. Cells were washed five times with PBS and left with 25 μl of PBS on fixed and stained cells and plates sealed with foil seal for image acquisition and stored at 4 °C.

### Image acquisition and analysis

Images of insoluble tau pathology and neuronal health markers were acquired by high-content microscopy on a cell imager (GE Healthcare, IN Cell Analyzer 2200) coupled with a robotic plate handler (PAA, S-Lab). For insoluble tau, four images were acquired per well with 10x magnification and 400 ms exposure time in the 488 nm channel and 30 ms exposure time for DAPI. For cell health markers, four images were acquired per well with 10x magnification and exposure times of 300 ms in the 488 nm (NeuN) channel, 300 ms in the 594 nm (MAP2) channel, and 30 ms for DAPI. Images were analyzed using IN Cell Investigator Developer Toolbox software (GE Healthcare). For tau pathology, we applied a detection threshold of 5000 relative fluorescence units (RFU) and a size exclusion sieve to exclude objects greater than 250 μm^2^ in the 488 nm channel and quantified the signal intensity, area, and intensity × area. The DAPI channel was quantified by applying object segmentation and size exclusion criteria of 50 to 1000 μm^2^ applied to quantify a count of DAPI-positive nuclei per image. For MAP2 staining, we applied a detection threshold of 3000 RFU with a maximum threshold of 65,535 RFU (maximal pixel intensity) in the 594 nm channel and quantified the signal intensity, area, and intensity × area. For NeuN, we applied a detection threshold of 4000 RFU with a maximum threshold of 65,535 RFU. We applied a size exclusion filter to exclude objects outside the range of 50 and 1000 μm^2^ and quantified the number of NeuN positive nuclei per image. DAPI was quantified as described above. The sum of all four images per well was used for each analysis.

### Analysis of immunocytochemistry data

Pathological tau was analyzed by normalizing the T49 staining intensity × area divided by the number of DAPI-positive cells per well. The negative controls were wells not receiving AD-tau (NT) and positive controls consisted of AD-tau fibril-treated (FT) wells in the absence of any test compound. The Z′ score was calculated for each plate based on the mean (μ) and standard deviation (σ) of 24 wells each of the negative and positive controls, utilizing the following equation:

$$Z^{\prime }=1-\frac{\left(3\sigma_{FT}+3\sigma_{NT}\right)}{\left(\mu_{FT}-\mu_{NT}\right)}$$

In this equation, FT refers to wells treated with AD-tau that form neuronal tau inclusions, whereas NT refers to wells that did not receive AD-tau and thus do not form neuronal tau inclusions. The T49 intensity × area normalized to DAPI counts was converted to percent inhibition for each compound using the equation:

$$\%\;Inhibition=\left(1-\frac{\left(\mu_{compound}-\mu_{NT}\right)}{\left(\mu_{FT}-\mu_{NT}\right)}\right)\times 100$$

Where μ refers to the mean normalized T49 value, compound refers to wells receiving AD-tau and test compound, NT refers to wells that received vehicle and no AD-tau, and FT refers to wells treated with AD-tau and vehicle.

### Biochemical extraction of neuron cultures for measurement of multimeric or monomeric tau

Media was aspirated from DIV21 neuron cultures treated with AD-tau as described above treated with or without compounds in 96-well plates and cells were washed three times with 150 μl of PBS. Subsequently, 50 μl of cold RIPA buffer comprised of 50 mM Tris pH 8.0, 150 mM sodium chloride, 5 mM EDTA, 0.5% sodium deoxycholate (Sigma-Aldrich), 1% NP-40 (Sigma-Aldrich), and 0.1% Sodium dodecyl sulfate (SDS, Fisher) supplemented with 1:100 phosphatase inhibitor and 1:1000 protease inhibitors, prepared as described previously, was added to each well and plates were incubated on ice for 10 min prior to cell lysis. Cells were lysed in RIPA buffer, with mixing *via* repeated pipetting to thoroughly lyse all cells in each well, with the collection of the cell lysates.

### Total protein assay

Neuronal culture lysates prepared as above were analyzed for total protein content, with 5 μl cell lysate diluted with 95 μl of a 1:50 mixture of BCA protein assay reagent A (Pierce) and BCA protein assay reagent B (Pierce) in clear flat-bottom 384-well plates (Thermo Fisher Scientific). The plate was covered and incubated 37 °C for 30 min, followed by determination of absorbance at 562 nm on a plate reader (Molecular Devices, SpectraMax M5). Total protein was calculated by extrapolation from a standard curve prepared with bovine serum albumin (BSA, Thermo Fisher Scientific).

### Rodent tau multimer ELISA

The rodent tau-specific antibody, mTau8, (a kind gift from Janssen Pharmaceuticals) was diluted to 2.5 μg/ml in 100 mM sodium carbonate pH 9.6 (Sigma-Aldrich), with 30 μl added per well in MaxiSorp 384-well plates (Thermo Fisher Scientific). Plates were centrifuged at 1000*g* for 1 min and stored at 4 °C overnight. Antibody-coated plates were washed five times with PBS containing 0.1% Tween20 (PBST, Sigma-Aldrich), and then blocked with the addition of 90 μl per well of heat-inactivated blocking buffer consisting of 1% BlockAce powder (BioRad) and 0.05% sodium azide (Sigma-Aldrich) in PBST. Plates were then incubated at 4 °C at least 4 days prior to use in ELISA analyses. For ELISA, plates were washed five times with PBST, and 30 μl of neuronal culture lysate diluted 5-fold in 0.2% BSA in PBS was added to wells and plates were incubated at 4 °C overnight. For use as a detection antibody, mTau8 was biotinylated (Thermo Fisher Scientific) and diluted to 0.2 mg/ml in Buffer C comprised of 20 mM sodium phosphate pH 7.0 (Sigma-Aldrich), 2 mM EDTA, 400 mM sodium chloride, 1% BSA and 0.005% Thimerosal (Thermo Fisher Scientific). For ELISA, antibody-coated plates that had incubated with neuronal culture lysates were washed five times in PBST, and 30 μl of biotinylated mTau8 in Buffer C was added to each well and incubated 37 °C for 1 h. Plates were washed five times in PBST and 30 μl of streptavidin-HRP (Thermo Scientific) diluted 1:10,000 in Buffer C was added to each well and incubated 37 °C for 1 h. Plates were then washed five times in PBST followed by addition to each well of 30 μl of a 1:1 mixture of TMB peroxidase substrate (SeraCare) and peroxidase substrate solution B (SeraCare). After incubation for 5 to 8 min at 22 °C to allow colorimetric development, the reaction was quenched with addition of 30 μl of 10% phosphoric acid (Fisher), and the absorbance at 450 nm was measured on a plate reader (Molecular Devices, SpectraMax M5).

### Rodent tau monomer ELISA

The Tau5 antibody (Covance) was diluted to 5 μg/ml in 100 mM sodium carbonate pH 9.6 (Sigma-Aldrich) with 30 μl added per well in MaxiSorp 384-well plates as described above for the rodent tau multimer ELISA. Plate washing and blocking were as described for the rodent tau multimer ELISA. Neuron lysates were prepared as described above from DIV21 neurons that were treated compounds but not AD-tau. Biotinylated mTau8, prepared as described above, was used as the detection antibody. For ELISA, antibody-coated plates that had incubated with neuronal culture lysates were washed five times in PBST, and 30 μl of biotinylated mTau8 in Buffer C was added to each well and incubated at 37 °C for 1 h. All subsequent ELISA steps were as described above for the rodent tau multimer ELISA.

### Immunoblot assessment of soluble and insoluble neuronal tau

Rat cortical neuronal cultures plated in 12-well plates that were treated with vehicle, BACE1 or γ-secretase inhibitors, as well as AD-tau, at DIV7 underwent cell extraction at DIV21. Media was aspirated and wells were washed with 1 ml of ice-cold PBS two times and then were lysed using 80 μl of ice-cold RIPA supplemented with protease inhibitor cocktail at 1:1000, phosphatase inhibitor cocktail at 1:100, and PMSF at 1:5000 (buffer components described in other methods above). After addition, each well was scraped with a 11 mm cell scraper (CytoOne) to recover all cellular material. Lysates were collected and sonicated with ten pulses on setting 2 using a handheld sonicator (Branson XL-2000 series). Lysate total protein concentration was determined using a BCA assay as described above. For the determination of soluble and insoluble total tau and phospho-tau species, homogenates were obtained from AD-tau-treated neuronal cultures with BACE1 or γ-secretase inhibitors, or vehicle only. Homogenates from identically treated triplicate wells from the same 12-well plate were pooled together as one sample and then subjected to centrifugation at 100,000*g* for 30 min. The supernatant fraction was used to assess soluble tau species, whereas the pellet was suspended in RIPA at ¼ of the total supernatant volume for assessment of insoluble tau species. The total protein concentration in the supernatant fraction was determined by BCA, and 20 μg of total protein underwent SDS-PAGE on 10% acrylamide gels of 1.5 mm thickness, run at 100 V for 90 min, followed by transfer onto nitrocellulose membranes, as previously described (50). A volume of the pellet fraction equal to that used to analyze the soluble fraction was also analyzed by SDS-PAGE followed by transfer to nitrocellulose membranes. Total rat tau was determined by immunostaining the nitrocellulose blots with the T49 antibody (1:1000 dilution from ascites), whereas the AT8 (0.1 μg/ml; Thermo Fisher), AT180 (0.1 μg/ml; Thermo Fisher) and PHF1 phospho-tau antibodies (1:5000 dilution from IgG preparation; kind gift from Dr Peter Davies) were used to detect pSer202/pThr205, pThr231 and pS396/pS404 tau, respectively. Secondary antibodies, imaging, and analysis were as previously described (50) using a LiCor scanner, with all quantified lanes within the detection limit of the instrument. All quantified tau bands (T49, AT8, AT180, and PHF1) from the supernatant fractions were normalized to the corresponding GAPDH integrated value. In the case of insoluble tau species, normalization was to the total GAPDH signal from the added supernatant and pellet fractions, with 25% of the pellet fraction GAPDH signal utilized to compensate for differences between the supernatant and pellet fraction volumes.

### Statistics

Compounds were screened in triplicate and the mean and standard deviations of negative and control wells (n = 24) were used to calculate the Z′-score for each screening plate, as described above. For concentration-response testing, compounds were analyzed in triplicate at each concentration and the mean and standard error of the mean are shown in graphs. For the determination of significance between multiple treatment groups, a one-way ANOVA with Dunnett’s multiple comparison tests was typically utilized, whereas a two-tailed *t* test was used for comparison of two groups. For non-parametric analyses, a Kruskal-Wallis test was used for multiple comparisons and a Mann–Whitney *U* test for the comparison of two groups.

## Data availability

Extensive study data are included in the Supporting information and additional data will be made available upon reasonable request to the corresponding author.

## Supporting information

This article includes supporting information.

## Conflict of interest

The authors declare that they have no conflicts of interest with the contents of this article.
