# Supplementary material for: Identification of small molecules and related targets that modulate tau pathology in a seeded primary neuron model
Source: J Biol Chem. 2023 Jun 1;299(7):104876. doi: 10.1016/j.jbc.2023.104876 (PMC10331484; doi:10.1016/j.jbc.2023.104876)

Supporting Information

Identification of small molecules and related targets that modulate tau pathology in a seeded primary neuron model

Garrett S. Gibbons[1,#], Hailey Gould[1,#], Virginia M-Y Lee[1], Alex Crowe[1] and Kurt R. Brunden[1,*]

[1]Center for Neurodegenerative Disease Research, Department of Pathology and Laboratory Medicine, Perelman School of Medicine, University of Pennsylvania, 3600 Spruce St., Philadelphia PA, 19104 USA

*Corresponding author

Address:

3600 Spruce St., 1 Maloney

Philadelphia, PA 19104

Email: kbrunden@upenn.edu

Telephone: (215) 615-5262

Table S1. List of 173 confirmed hits that caused ≥30% reduction neuronal tau pathology and ≥30% inhibition of tau multimers. The results are provided as % reduction of T49 immunocytochemical staining observed in the primary (1^0) rat cortical neuron screen, in the repeat confirmation activity analyses conducted in parallel to neurotoxicity testing, and in the orthogonal mTau8 tau oligomer ELISA (mean and standard deviation for each). For each compound, the catalog number from the vendor (Cat #), the compound name, and the supplied annotation on putative target(s) and pathway(s) are supplied.

Cat #	Compound Name	Putative Target	Putative Pathway	1^0 Screen T49 ICC %Inh	SD	Repeat T49 ICC %Inh	SD	mTau8 ELISA %Inh	SD
HY-N0521A	(+)-Gallocatechin	Others	Others	56.2	3.6	87.9	0.4	59.9	8.8
HY-N0172	Caffeic acid	Endogenous Metabolite; Lipoxygenase; TRP Channel	Membrane Transporter/Ion Channel; Metabolic Enzyme/Protease; Neuronal Signaling	58.9	11.5	80.0	7.6	70.6	2.2
HY-13519	TRAM-34	Potassium Channel	Membrane Transporter/Ion Channel	41.4	4.5	77.9	2.5	55.0	11.6
HY-15456	NVP-BVU972	c-Met/HGFR	Protein Tyrosine Kinase/RTK	59.4	11.4	75.0	8.0	63.4	1.9
HY-100431	IMR-1	Notch	Neuronal Signaling; Stem Cell/Wnt	63.3	13.1	70.4	15.6	34.1	2.4
HY-79511	FAAH-IN-2	Autophagy; FAAH	Autophagy; Metabolic Enzyme/Protease; Neuronal Signaling	44.3	6.1	67.7	1.5	49.0	5.0
HY-14450	JNJ-31020028	Neuropeptide Y Receptor	GPCR/G Protein; Neuronal Signaling	45.6	11.5	66.1	3.6	47.0	6.3
HY-B0105B	(-)-Ketoconazole	Fungal	Anti-infection	44.1	12.2	65.4	3.9	48.9	5.2
HY-12273	DMH-1	Autophagy; TGF-β Receptor	Autophagy; TGF-beta/Smad	32.5	10.3	62.0	7.0	65.1	11.5
HY-13422A	Zatebradine	HCN Channel	Membrane Transporter/Ion Channel	44.7	11.2	55.1	2.2	37.7	8.2
HY-16207	FGH10019	Fatty Acid Synthase (FASN)	Metabolic Enzyme/Protease	46.6	7.2	53.5	16.6	44.5	4.9
HY-N0898	Catechin	Apoptosis; COX; Endogenous Metabolite; Influenza Virus	Anti-infection; Apoptosis; Immunology/Inflammation; Metabolic Enzyme/Protease	36.2	9.6	52.8	6.1	31.2	0.7
HY-B0553	Methazolamide	Carbonic Anhydrase	Metabolic Enzyme/Protease	36.4	24.9	52.4	14.7	47.5	9.2
HY-18681	Voxelotor	Others	Others	43.3	2.6	51.8	10.9	39.4	10.2
HY-13422	Zatebradine (hydrochloride)	HCN Channel	Membrane Transporter/Ion Channel	49.5	3.6	49.4	12.9	33.9	16.4
HY-13288	Org 27569	Cannabinoid Receptor	GPCR/G Protein; Neuronal Signaling	43.7	15.3	41.8	9.7	53.9	9.0
HY-18700	BRD73954	HDAC	Cell Cycle/DNA Damage; Epigenetics	35.7	17.7	38.1	2.8	37.5	1.8

HY-12591	D-Luciferin (sodium salt)	Others	Others	45.3	31.2	34.6	6.2	36.8	6.0
HY-B0992	Nithiamide	Antibiotic; Bacterial	Anti-infection	76.6	5.5	82.8	1.8	56.2	0.5
HY-N2117	Isoginkgetin	MMP	Metabolic Enzyme/Protease	48.1	10.9	60.7	4.6	44.7	15.2
HY-122856	AZ12601011	TGF-β Receptor	TGF-beta/Smad	47.2	8.1	59.3	4.8	46.2	0.2
HY-22385	Salsolidine	Monoamine Oxidase	Neuronal Signaling	52.1	6.9	54.1	12.1	45.1	8.6
HY-15717	kobe2602	Ras	GPCR/G Protein	42.8	3.5	30.7	26.0	47.6	0.4
HY-59090	1-Azakenpaullone	GSK-3	PI3K/Akt/mTOR; Stem Cell/Wnt	79.4	1.9	80.5	2.0	61.9	2.4
HY-N0236	Corylin	Antibiotic; Bacterial	Anti-infection	44.0	6.9	75.4	4.2	47.6	3.3
HY-18938	Selonsertib	Apoptosis; MAP3K	Apoptosis; MAPK/ERK Pathway	73.1	5.5	72.2	7.8	51.5	0.9
HY-19834	Fenebrutinib	Btk	Protein Tyrosine Kinase/RTK	61.7	6.7	67.8	1.2	51.1	5.8
HY-19867A	Burixafor (hydrobromide)	CXCR	GPCR/G Protein; Immunology/Inflammation	56.7	3.1	66.0	9.2	33.9	6.9
HY-14151	Prucalopride	5-HT Receptor	GPCR/G Protein; Neuronal Signaling	59.9	6.8	55.3	1.3	34.8	5.0
HY-N0236	Corylin	Antibiotic; Bacterial	Anti-infection	44.0	6.9	53.9	20.0	46.8	6.6
HY-129974	3,3'-Diiodo-L-thyronine	COX; Endogenous Metabolite	Immunology/Inflammation; Metabolic Enzyme/Protease	40.9	12.8	49.6	5.1	35.9	0.3
HY-N0580	Fraxetin	Apoptosis	Apoptosis	51.6	17.6	48.9	5.8	62.8	0.9
HY-W027968	Coumarin-3-carboxylic Acid	Others	Others	50.0	4.3	48.2	12.2	30.5	7.9
HY-112613	UCB9608	PI4K	PI3K/Akt/mTOR	44.0	6.9	67.8	8.4	52.0	9.3
HY-13719	Oleandrin	Na+/K+ ATPase	Membrane Transporter/Ion Channel	50.4	13.5	61.8	9.1	60.6	3.6
HY-111680	PTI-428	Autophagy; CFTR	Autophagy; Membrane Transporter/Ion Channel	44.0	14.7	53.0	10.0	35.1	1.5
HY-126290	Tyk2-IN-8	JAK	Epigenetics; JAK/STAT Signaling; Stem Cell/Wnt	49.0	8.5	52.7	9.1	44.2	1.1
HY-114415	AWZ1066S	Parasite	Anti-infection	52.3	24.2	65.6	5.6	41.2	8.9
HY-76612	SB-408124 (Hydrochloride)	Orexin Receptor (OX Receptor)	GPCR/G Protein; Neuronal Signaling	46.4	2.4	53.8	13.0	39.0	0.9
HY-14581	Palomid 529	Apoptosis; mTOR	Apoptosis; PI3K/Akt/mTOR	40.7	11.3	50.7	5.8	51.5	10.6
HY-N0885	Telocinobufagin	Others	Others	49.2	10.7	47.2	7.1	53.8	10.8
HY-B0275	Oxytetracycline	Antibiotic; Bacterial; Endogenous Metabolite; HSV	Anti-infection; Metabolic Enzyme/Protease	50.6	9.1	45.6	7.2	31.1	3.6

HY-N0006	Demethoxycurcumin	Apoptosis; Autophagy; Bacterial	Anti-infection; Apoptosis; Autophagy	46.1	8.6	44.0	4.7	38.5	0.2
HY-15951	ML167	CDK	Cell Cycle/DNA Damage	63.4	8.8	40.0	9.7	30.1	1.1
HY-N0923	Corydaline	AChE	Neuronal Signaling	37.9	32.7	34.9	4.3	34.0	6.9
HY-B0204	Pimobendan	Phosphodiesterase (PDE)	Metabolic Enzyme/Protease	43.7	4.2	33.6	22.3	45.1	7.0
HY-13600	Clobetasol propionate	Glucocorticoid Receptor	GPCR/G Protein	48.3	5.4	32.3	7.2	32.8	12.8
HY-17592A	Bithionol (sulfoxide)	Parasite	Anti-infection	41.1	11.0	77.8	21.4	56.0	8.5
HY-B0557	Bisacodyl	Others	Others	63.4	15.7	39.1	11.3	36.0	5.3
HY-N7066	Difloxacin (hydrochloride)	Antibiotic; Bacterial	Anti-infection	33.6	30.7	36.5	10.4	37.7	5.4
HY-10342	Enzastaurin	Apoptosis; Autophagy; PKC	Apoptosis; Autophagy; Epigenetics; TGF-beta/Smad	90.9	0.9	86.2	2.7	81.4	0.6
HY-17510	Gossypol (acetic acid)	Bcl-2 Family	Apoptosis	67.2	11.4	71.7	12.6	60.5	11.3
HY-N0284	Esculetin	Akt; PI3K	PI3K/Akt/mTOR	79.4	4.6	68.8	6.1	50.7	4.2
HY-B0371B	(R)-Terazosin	Adrenergic Receptor	GPCR/G Protein; Neuronal Signaling	69.4	9.0	67.1	6.8	53.6	0.2
HY-16425	RG2833	HDAC	Cell Cycle/DNA Damage; Epigenetics	43.6	20.3	64.6	6.4	64.3	19.1
HY-19836	PF06650833	IRAK	Immunology/Inflammation	68.2	8.2	60.4	10.9	69.2	2.7
HY-12409	PFI-3	Epigenetic Reader Domain	Epigenetics	61.0	4.2	56.9	11.4	52.5	5.1
HY-100740C	(1α,1'S,4β)-Lanabecestat	Beta-secretase	Neuronal Signaling	59.1	7.0	56.5	7.9	47.4	4.2
HY-50858	Ruxolitinib (phosphate)	Autophagy; JAK; Mitophagy	Autophagy; Epigenetics; JAK/STAT Signaling; Stem Cell/Wnt	57.5	20.9	55.7	11.5	71.7	1.7
HY-12013	PD153035 (Hydrochloride)	EGFR	JAK/STAT Signaling; Protein Tyrosine Kinase/RTK	58.5	9.9	55.3	2.8	71.9	4.8
HY-12323	ISX-9	Others	Others	43.8	11.6	52.4	3.5	54.3	8.0
HY-16558	Butein	Apoptosis; Autophagy; EGFR; Phosphodiesterase (PDE)	Apoptosis; Autophagy; JAK/STAT Signaling; Metabolic Enzyme/Protease; Protein Tyrosine Kinase/RTK	56.4	9.6	50.9	18.6	54.1	4.8
HY-N0117	Indirubin	Apoptosis	Apoptosis	70.3	6.9	50.3	5.2	54.4	17.7
HY-15298	Grazoprevir	HCV; HCV Protease	Anti-infection; Metabolic Enzyme/Protease	50.6	6.8	49.9	67.6	40.1	0.1
HY-100675	JTE-013	Apoptosis; LPL Receptor	Apoptosis; GPCR/G Protein	60.0	10.3	49.8	18.9	34.5	4.2
HY-117626	LP-935509	Others	Others	31.7	3.2	45.7	18.9	66.4	11.5

HY-A0022A	Azaphen (dihydrochloride monohydrate)	Serotonin Transporter	Neuronal Signaling	52.5	8.7	45.5	11.4	38.6	5.7
HY-N0621	Morin	Others	Others	42.8	4.7	44.1	17.0	70.3	4.3
HY-N0690	Schisandrin C	Apoptosis; Virus Protease	Anti-infection; Apoptosis	37.3	19.2	43.8	18.7	30.3	10.3
HY-50098A	Mardepodect (hydrochloride)	Phosphodiesterase (PDE)	Metabolic Enzyme/Protease	58.9	2.8	43.0	4.9	37.0	9.9
HY-N0219	(+)-Bicuculline	GABA Receptor	Membrane Transporter/Ion Channel; Neuronal Signaling	42.5	14.4	39.9	8.4	43.1	8.8
HY-W017113	2-Mercaptobenzothiazole	Endogenous Metabolite	Metabolic Enzyme/Protease	35.5	14.9	37.6	1.9	58.1	3.0
HY-10069	Y-33075 (dihydrochloride)	ROCK	Cell Cycle/DNA Damage; Cytoskeleton; Stem Cell/Wnt; TGF-beta/Smad	44.9	3.6	33.8	17.3	76.4	2.2
HY-17511	Potassium oxonate	Others	Others	42.1	9.3	32.4	19.2	52.9	4.3
HY-113016	Elaidic acid	Endogenous Metabolite	Metabolic Enzyme/Protease	46.4	7.2	31.2	13.8	53.4	7.4
HY-N0570	Hydroxytyrosol	Endogenous Metabolite	Metabolic Enzyme/Protease	82.1	2.8	82.8	3.0	76.7	3.1
HY-16680	Helioxanthin 8-1	HBV	Anti-infection	69.8	6.6	71.9	3.2	87.4	0.4
HY-N0656A	(+)-Usnic acid	mTOR	PI3K/Akt/mTOR	62.6	6.1	65.3	2.1	71.5	2.0
HY-B0371D	(S)-Terazosin	Adrenergic Receptor	GPCR/G Protein; Neuronal Signaling	63.8	3.3	65.2	5.9	60.3	4.2
HY-N0696	Sipeimine	Others	Others	59.8	9.9	64.7	8.6	57.1	8.0
HY-N0776	Isorhamnetin	Endogenous Metabolite; MEK; PI3K	MAPK/ERK Pathway; Metabolic Enzyme/Protease; PI3K/Akt/mTOR	47.2	11.1	62.4	9.8	68.3	5.6
HY-D0254	Gallein	Others	Others	63.6	4.1	60.6	5.0	65.1	5.4
HY-18733	Lipoic acid	Endogenous Metabolite; Mitochondrial Metabolism; Reactive Oxygen Species	Immunology/Inflammation; Metabolic Enzyme/Protease; NF-κB	61.8	6.1	59.9	3.5	71.2	6.9
HY-10472	LY2811376	Beta-secretase	Neuronal Signaling	50.4	7.0	58.2	6.3	62.2	4.6
HY-123963	C-178	STING	Immunology/Inflammation	59.9	3.1	53.5	8.2	54.1	5.5
HY-N0018	Daidzin	Mitochondrial Metabolism; Reverse Transcriptase	Anti-infection; Metabolic Enzyme/Protease	37.7	14.7	52.6	10.0	76.6	12.7
HY-N0602	Ginsenoside Rg2	Amyloid-β; NF-κB	Neuronal Signaling; NF-κB	51.3	14.6	52.6	5.4	45.5	4.3
HY-12824	RNPA1000	Antibiotic; Bacterial	Anti-infection	54.7	11.4	51.8	10.5	62.2	7.8
HY-116677	Tris(benzyltriazolylmethyl)amine	Others	Others	47.7	4.3	48.9	5.0	57.3	2.9

HY-101855	Anle138b	Others	Others	47.6	4.3	46.0	1.6	71.8	0.9
HY-120934	C25-140	E1/E2/E3 Enzyme; TNF Receptor	Apoptosis; Metabolic Enzyme/Protease	45.1	4.3	42.1	19.2	58.5	0.8
HY-N0121	Sesamin	Others	Others	41.3	15.2	40.5	5.2	43.6	1.0
HY-N0880	Cinobufotalin	Others	Others	46.0	2.2	39.7	3.7	71.8	2.9
HY-B1016	Trapidil	PDGFR	Protein Tyrosine Kinase/RTK	41.0	13.0	33.2	14.4	44.4	1.1
HY-D0086	DIDS (sodium salt)	Others	Others	80.0	5.2	78.3	7.3	69.0	8.3
HY-114180	RU.521	Others	Others	34.1	13.2	57.4	4.3	31.5	14.2
HY-N0400	Wogonin	Apoptosis; Autophagy; CDK; Wnt	Apoptosis; Autophagy; Cell Cycle/DNA Damage; Stem Cell/Wnt	49.5	8.6	56.1	8.1	55.0	12.4
HY-112667	CU-CPT-9a	Toll-like Receptor (TLR)	Immunology/Inflammation	49.5	3.3	44.0	2.9	42.8	20.0
HY-107854	N-Acetyl-5-hydroxytryptamine	Endogenous Metabolite; Trk Receptor	Metabolic Enzyme/Protease; Neuronal Signaling; Protein Tyrosine Kinase/RTK	40.3	8.3	41.9	6.0	64.9	6.5
HY-N1949	Homoplantaginin	NF-κB; TNF Receptor	Apoptosis; NF-κB	53.7	4.2	38.0	18.9	51.1	18.6
HY-108915	Trimethylamine N-oxide (dihydrate)	Endogenous Metabolite	Metabolic Enzyme/Protease	65.9	13.5	34.3	4.2	38.0	15.8
HY-14608A	L-Glutamic acid monosodium salt	Apoptosis; Ferroptosis; iGluR	Apoptosis; Membrane Transporter/Ion Channel; Neuronal Signaling	90.9	2.8	82.9	7.0	70.8	7.5
HY-15427	GDC-0834	Btk	Protein Tyrosine Kinase/RTK	73.5	8.6	66.0	5.3	39.5	2.4
HY-B1452	Licofelone	Apoptosis; COX; Lipoxygenase	Apoptosis; Immunology/Inflammation; Metabolic Enzyme/Protease	31.1	19.4	59.1	13.4	65.9	7.4
HY-123999	CD38 inhibitor 1	Others	Others	48.4	11.7	56.7	12.9	43.9	8.0
HY-75308	Azetidine-2-carboxylic acid	Others	Others	60.5	2.0	52.9	6.4	50.7	3.5
HY-B0455	Lomefloxacin (hydrochloride)	Antibiotic; Bacterial	Anti-infection	40.8	1.5	42.0	9.8	77.1	26.3
HY-13282	GANT 58	Gli	Stem Cell/Wnt	57.9	4.7	34.9	5.7	51.4	0.4
HY-106376	D,L-Buthionine-(S,R)-sulfoximine	Ferroptosis	Apoptosis	36.8	7.8	32.1	4.6	31.1	8.0
HY-19752A	VU0357017 (hydrochloride)	mAChR	GPCR/G Protein; Neuronal Signaling	31.7	2.3	30.5	10.8	49.5	12.7
HY-15681	Senexin A	CDK	Cell Cycle/DNA Damage	55.9	18.6	85.5	1.5	69.8	4.3
HY-101736	AMG9810	TRP Channel	Membrane Transporter/Ion Channel; Neuronal Signaling	44.2	29.0	73.7	11.1	53.3	7.6
HY-100792	URB602	Others	Others	46.5	13.2	63.1	5.9	39.3	14.2

HY-B1438	Canrenone	Endogenous Metabolite; Mineralocorticoid Receptor	Metabolic Enzyme/Protease	40.4	15.9	58.5	10.1	48.0	5.8
HY-15068	NBQX	iGluR	Membrane Transporter/Ion Channel; Neuronal Signaling	37.5	16.4	55.8	1.1	31.4	15.8
HY-18976	UF010	HDAC	Cell Cycle/DNA Damage; Epigenetics	57.0	3.9	48.8	16.0	39.7	1.4
HY-112776	BN82002	Phosphatase	Metabolic Enzyme/Protease	34.7	10.1	45.0	7.8	43.0	5.4
HY-15124	(S)-(-)-Bay-K-8644	Calcium Channel	Membrane Transporter/Ion Channel; Neuronal Signaling	51.3	7.8	43.9	9.2	39.7	8.3
HY-112629	PDM2	Aryl Hydrocarbon Receptor	Immunology/Inflammation	42.6	16.9	34.2	22.8	30.9	16.8
HY-13240	LY2886721	Beta-secretase	Neuronal Signaling	32.1	1.7	58.8	23.4		
HY-B1153	Glafenine	Others	Others	34.3	6.5	65.2	2.6	72.0	2.8
HY-P0018A	Pepstatin Trifluoroacetate	HIV Protease; Proteasome	Anti-infection; Metabolic Enzyme/Protease	66.8	5.9	70.0	2.2	64.4	4.4
HY-N2022	Castanospermine	Glucosidase	Metabolic Enzyme/Protease	61.7	5.9	68.0	6.7	69.3	7.5
HY-19995	GSK137647A	GPR120	GPCR/G Protein	45.4	13.2	64.9	8.7	53.3	2.8
HY-135652	Hexyl gallate	Parasite	Anti-infection	63.4	2.0	66.8	9.3	76.9	6.5
HY-129701	MCL-1/BCL-2-IN-3	Bcl-2 Family	Apoptosis	46.1	8.8	46.8	10.4	76.0	1.5
HY-N0382	Galangin	Autophagy; Cytochrome P450	Autophagy; Metabolic Enzyme/Protease	59.1	2.0	34.5	13.0	80.1	7.7
HY-12477	PF-06447475	LRRK2	Autophagy	47.3	17.8	59.9	4.6	68.3	4.7
HY-11010	AS601245	JNK	MAPK/ERK Pathway	67.4	19.7	89.9		62.6	53.7
HY-19562	PF-06260933	MAP4K	MAPK/ERK Pathway	72.3	1.5	88.3		48.1	10.2
HY-12787	L-779450	Autophagy; Raf	Autophagy; MAPK/ERK Pathway	74.4	1.4	85.1		52.8	8.3
HY-10295	SB 202190	Apoptosis; Autophagy; Ferroptosis; p38 MAPK	Apoptosis; Autophagy; MAPK/ERK Pathway	63.8	20.2	83.3		78.7	30.8
HY-N0162	Luteolin	Apoptosis; Autophagy; Endogenous Metabolite	Apoptosis; Autophagy; Metabolic Enzyme/Protease	60.9	2.8	78.9		79.4	5.0
HY-101611	MSC2530818	CDK	Cell Cycle/DNA Damage	72.5	3.4	78.8		89.2	5.2
HY-N1579	Pyrogallol	Apoptosis; Endogenous Metabolite; Fungal	Anti-infection; Apoptosis; Metabolic Enzyme/Protease	50.1	6.3	78.7		36.7	1.2
HY-N1913	Danshensu	Apoptosis; Autophagy; Keap1-Nrf2	Apoptosis; Autophagy; NF-κB	68.5	9.1	78.6		70.4	36.0

HY-101474A	Zanubrutinib	Btk	Protein Tyrosine Kinase/RTK	33.0	7.5	66.2		68.9	2.5
HY-15427B	GDC-0834 (S-enantiomer)	Btk	Protein Tyrosine Kinase/RTK	50.1	4.1	65.9		45.6	0.7
HY-50865	PDE-9 inhibitor	Phosphodiesterase (PDE)	Metabolic Enzyme/Protease	50.8	6.5	63.9		90.7	8.7
HY-100482	CPI-637	Epigenetic Reader Domain; Histone Acetyltransferase	Epigenetics	51.1	7.9	63.7		53.8	17.2
HY-100726	GNE-272	Epigenetic Reader Domain; Histone Acetyltransferase	Epigenetics	66.3	5.7	63.4		58.2	6.8
HY-B0795	MHY1485	Autophagy; mTOR	Autophagy; PI3K/Akt/mTOR	50.5	7.6	59.3		91.8	12.3
HY-B0797	Etretinate	Apoptosis	Apoptosis	60.7	8.5	56.7		34.2	22.0
HY-12594	Paritaprevir	HCV; HCV Protease	Anti-infection; Metabolic Enzyme/Protease	53.4	10.6	56.2		51.6	8.1
HY-100693	SYP-5	HIF/HIF Prolyl-Hydroxylase	Metabolic Enzyme/Protease	54.0	14.2	55.7		55.0	7.4
HY-13613	Dutasteride	5 alpha Reductase; Apoptosis	Apoptosis; Metabolic Enzyme/Protease	59.4	5.0	53.0		66.9	41.3
HY-101494	LY3214996	ERK	MAPK/ERK Pathway; Stem Cell/Wnt	52.9	8.8	51.5		44.4	5.6
HY-128583	G150	Others	Others	57.7	6.3	49.3		63.0	7.2
HY-10204	OSI-930	Apoptosis; c-Fms; c-Kit; VEGFR	Apoptosis; Protein Tyrosine Kinase/RTK	65.8	17.6	47.9		55.4	0.6
HY-N0453	Hypericin	Apoptosis; Influenza Virus	Anti-infection; Apoptosis	49.6	11.6	76.4		53.8	5.6
HY-10435A	SKF-82958 (hydrobromide)	Dopamine Receptor	GPCR/G Protein; Neuronal Signaling	44.3	2.6	68.8		67.8	4.4
HY-B0371A	Terazosin (hydrochloride dihydrate)	Adrenergic Receptor	GPCR/G Protein; Neuronal Signaling	43.0	2.9	68.6		67.3	2.0
HY-10473	Eprotirome	Thyroid Hormone Receptor	Others	34.8	7.6	67.8		43.6	3.5
HY-117535	CDK2-IN-4	CDK	Cell Cycle/DNA Damage	38.6	2.6	67.2		59.5	3.5
HY-W013372	7,8-Dihydroxyflavone	Apoptosis; Trk Receptor	Apoptosis; Neuronal Signaling; Protein Tyrosine Kinase/RTK	35.5	7.8	65.8		54.8	4.0
HY-14731	VE-821	ATM/ATR	Cell Cycle/DNA Damage; PI3K/Akt/mTOR	41.0	3.2	65.3		70.0	3.1
HY-76772	Cevimeline (hydrochloride hemihydrate)	mAChR	GPCR/G Protein; Neuronal Signaling	31.0	3.1	61.2		61.6	4.6

HY-70050C	Alosetron (Hydrochloride)	5-HT Receptor	GPCR/G Protein; Neuronal Signaling	34.8	26.8	60.4		56.8	0.4
HY-12949	ML204	TRP Channel	Membrane Transporter/Ion Channel; Neuronal Signaling	37.4	16.4	59.0		59.4	6.2
HY-114269	(-)-(S)-B-973B	nAChR	Membrane Transporter/Ion Channel; Neuronal Signaling	34.9	13.4	57.7		60.9	1.6
HY-B0443A	Xylazine (hydrochloride)	Adrenergic Receptor	GPCR/G Protein; Neuronal Signaling	38.2	6.0	56.0		46.5	11.2
HY-13406	TAK-779	CCR; CXCR; HIV	Anti-infection; GPCR/G Protein; Immunology/Inflammation	36.6	64.3	55.5		37.9	8.7
HY-12528	DBPR108	Dipeptidyl Peptidase	Metabolic Enzyme/Protease	42.8	9.0	54.3		58.8	7.1
HY-111354	Tinoridine hydrochloride	Glutathione Peroxidase	Metabolic Enzyme/Protease	32.8	8.0	54.2		65.6	6.3
HY-101027	GSK 4027	Epigenetic Reader Domain; Histone Acetyltransferase	Epigenetics	42.3	6.0	50.9		87.7	0.2
HY-112708A	PF-06700841 (P-Tosylate)	JAK	Epigenetics; JAK/STAT Signaling; Stem Cell/Wnt	42.1	8.0	49.3		31.0	0.7
HY-B0588	Brinzolamide	Carbonic Anhydrase	Metabolic Enzyme/Protease	35.5	5.9	49.1		44.4	1.9
HY-19764	GSK2983559 active metabolite	RIP kinase	Apoptosis	30.0	13.0	47.6		38.3	13.3
HY-13907	TCS 359	FLT3	Protein Tyrosine Kinase/RTK	37.0	22.2	47.0		47.0	0.8
HY-75054	Abiraterone (acetate)	Cytochrome P450	Metabolic Enzyme/Protease	56.2	16.1	64.7		59.6	7.6
HY-112910	Grp94 Inhibitor-1	HSP	Cell Cycle/DNA Damage; Metabolic Enzyme/Protease	61.1	12.8	60.1		84.0	2.6
HY-100351	BI-7273	Epigenetic Reader Domain	Epigenetics	51.5	6.2	55.3		67.5	1.9
HY-117650A	RG7834	HBV	Anti-infection	53.0	2.9	40.4		48.2	15.7

Table S2. Enhancers of neuronal tau inclusions. The results are provided as the mean % increase and standard deviation observed in the primary rat cortical neuron T49 immunocytochemical screen and in the orthogonal mTau8 tau oligomer ELISA. For each compound, the catalog number from the vendor (Cat #), the compound name, and the supplied annotation on putative target(s) and pathway(s) are supplied.

Cat #	Compound Name	Putative Target	Putative Pathway	T49 ICC % increase	SD	mTau8 ELISA % increase	SD
HY-121186	Bevantolol (hydrochloride)	Adrenergic Receptor; Calcium Channel	GPCR/G Protein; Membrane Transporter/Ion Channel; Neuronal Signaling	62.9	19.8	89.1	18.2
HY-B0381	Betaxolol	Adrenergic Receptor	GPCR/G Protein; Neuronal Signaling	51.8	20.5	43.2	4.0
HY-19756	OTX008	Galectin	Immunology/Inflammation	47.8	9.8	130.1	10.8
HY-B0949	Protriptyline (hydrochloride)	5-HT and noradrenaline reuptake; AChE	Neuronal Signaling	50.3	32.1	77.7	18.7
HY-15227	EPZ004777	Apoptosis; Histone Methyltransferase	Apoptosis; Epigenetics	88.0	38.5	96.6	12.8
HY-116790A	(+)-Penbutolol	Adrenergic Receptor	GPCR/G Protein; Neuronal Signaling	71.3	15.3	43.1	1.7
HY-B1213	Trimipramine (maleate)	5-HT and noradrenaline reuptake; 5-HT and Dopamine Receptors; Bacterial	Anti-infection; GPCR/G Protein; Neuronal Signaling	50.7	28.9	79.2	28.4
HY-125837A	MS31 (trihydrochloride)	Epigenetic Reader Domain	Epigenetics	86.8	8.4	124.9	5.1

Table S3. Triplicate retesting of γ-secretase inhibitors at 10 µM in the mTau8 rodent tau multimer ELISA reveals significant inhibition of neuronal multimeric tau species without meaningful evidence of toxicity as determined by % protein reduction in culture homogenates.

Cat #	Compound	% mTau8 Inhibition	% total protein reduction
HY-10009	Semagacestat	56.3 +/- 3.9	-2.6 +/-6.5
HY-12449	Crenigacestat	45.3 +/- 0.7	2.6 +/- 4.0
HY-13027	DAPT	75.7 +/- 2.6	-8.6 +/- 5.2

Figure S1. Extraction of pathological tau from AD patient tissue. **A**) Immunohistochemical staining with the PHF1 antibody to detect phosphorylated tau pathology in frontal cortex, demonstrating neurofibrillary tangles, neuritic plaque, and abundant neuropil thread tau pathology. **B**) Workflow for preparation of enriched insoluble AD-tau extracts. **C**) Western blot demonstrating the amount of total tau (K9JA antibody) and phospho-tau (PHF1 antibody) in the various fractions from B., including the Sark pellet 2 containing the final enriched AD-tau that is relatively devoid of contaminating proteins based on Ponceau S stain.

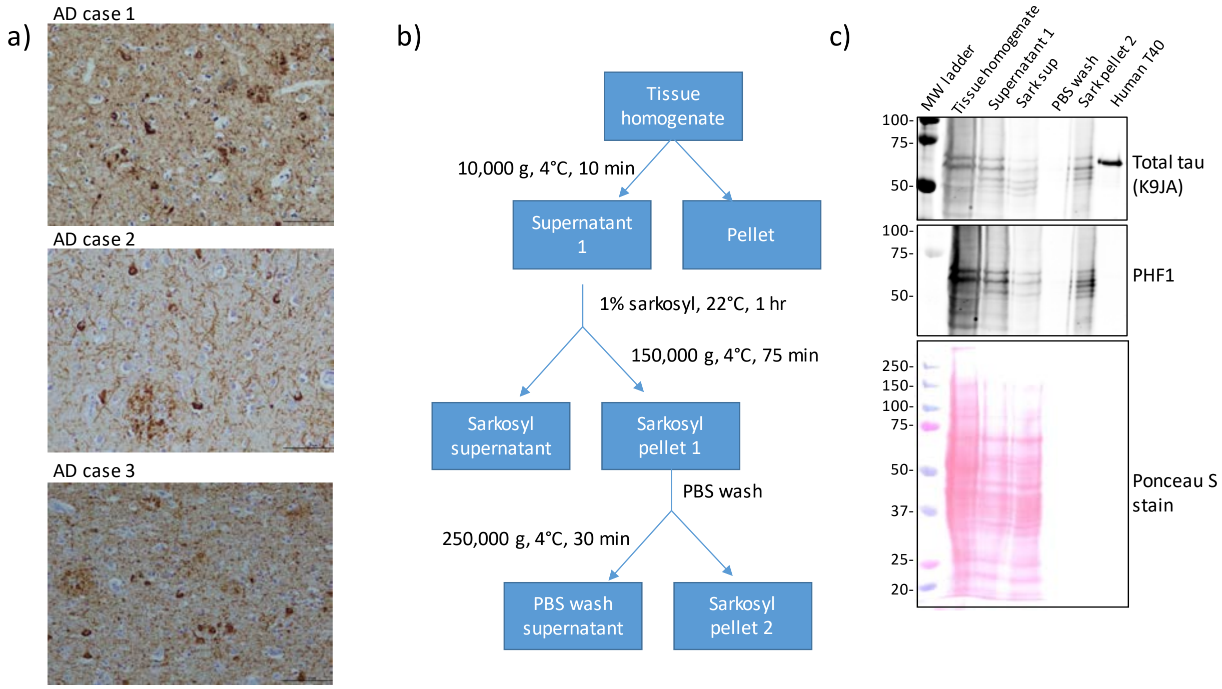

Figure S2. Characterization of primary rat cortical neuron cultures. Immunocytochemical staining of neuron markers NeuN and MAP2 at the time of compound treatment and AD-tau transduction (DIV 7; left panels) and at the time of tau pathology analysis (DIV 21; right panels). Staining with the astrocytic marker GFAP demonstrates that the majority of non-neuronal cells are astrocytes.

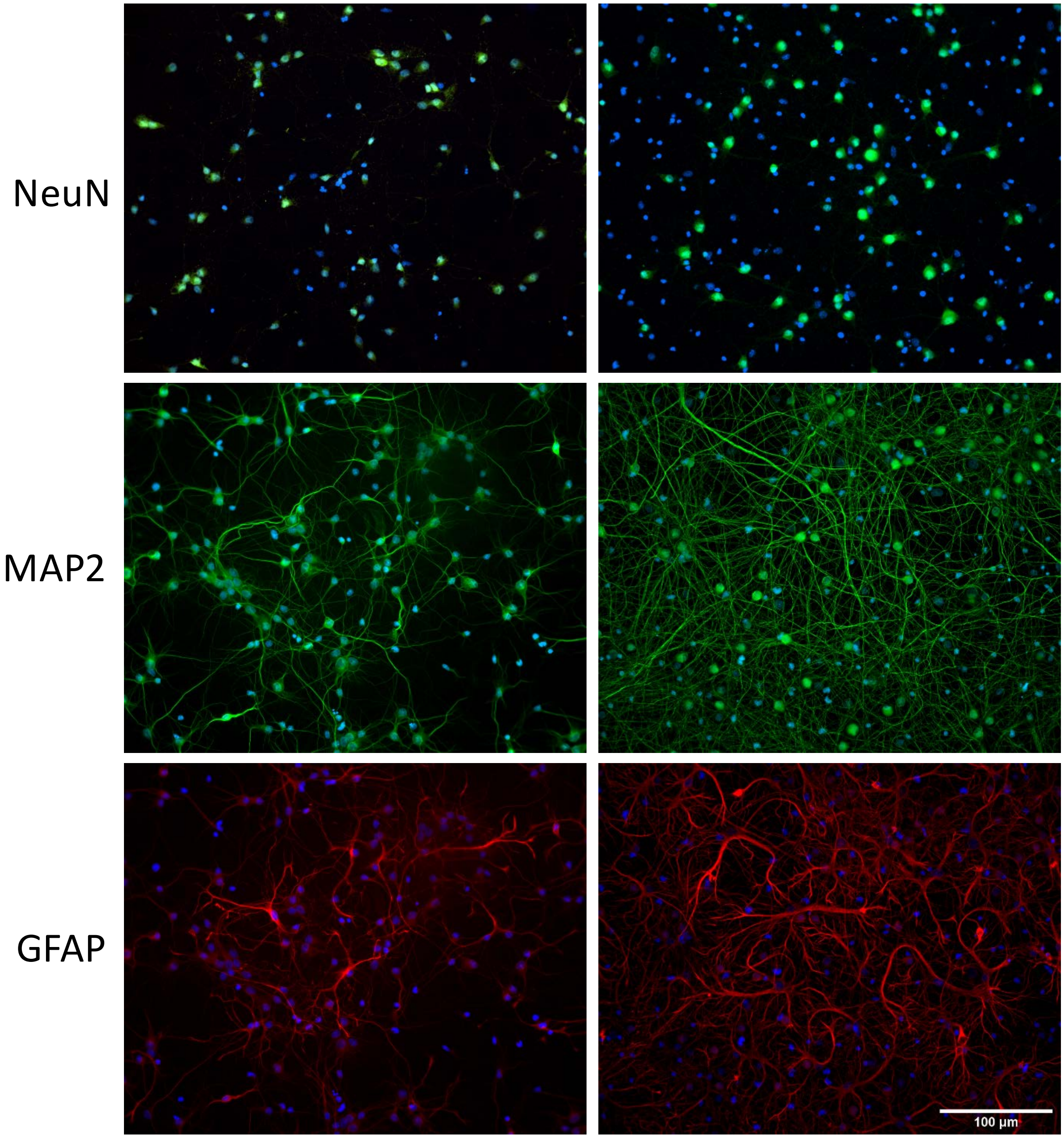

Figure S3. Concentration-response results of repurchased compounds in which modulation of neuronal tau was separated from measures of toxicity. Tau inclusions (green squares), total DAPI-positive cell counts (blue circles), NeuN-positive neuronal cell counts (black triangles) and MAP2-positive neuronal dendrites (red triangles) are plotted.

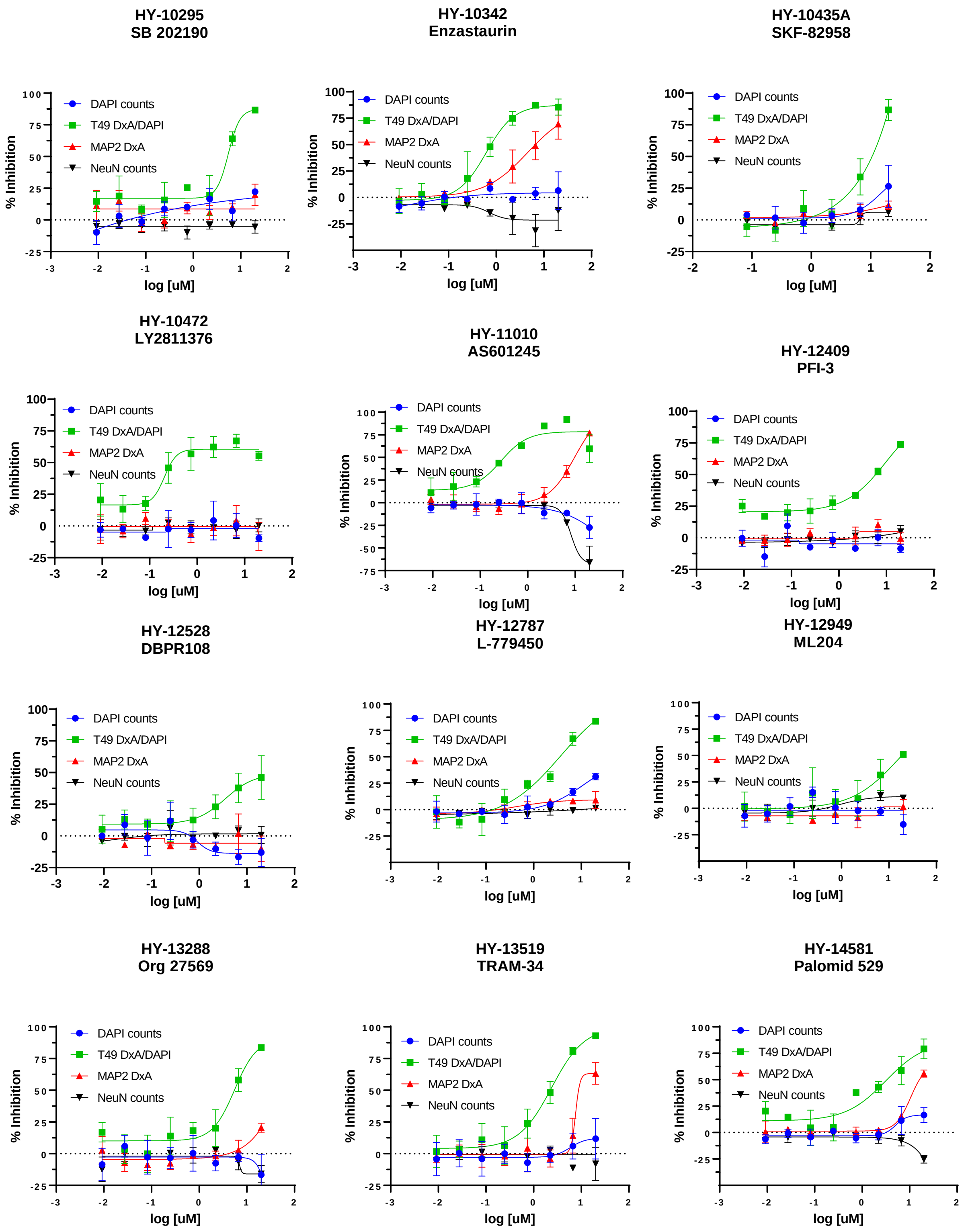

Figure S3. Continued

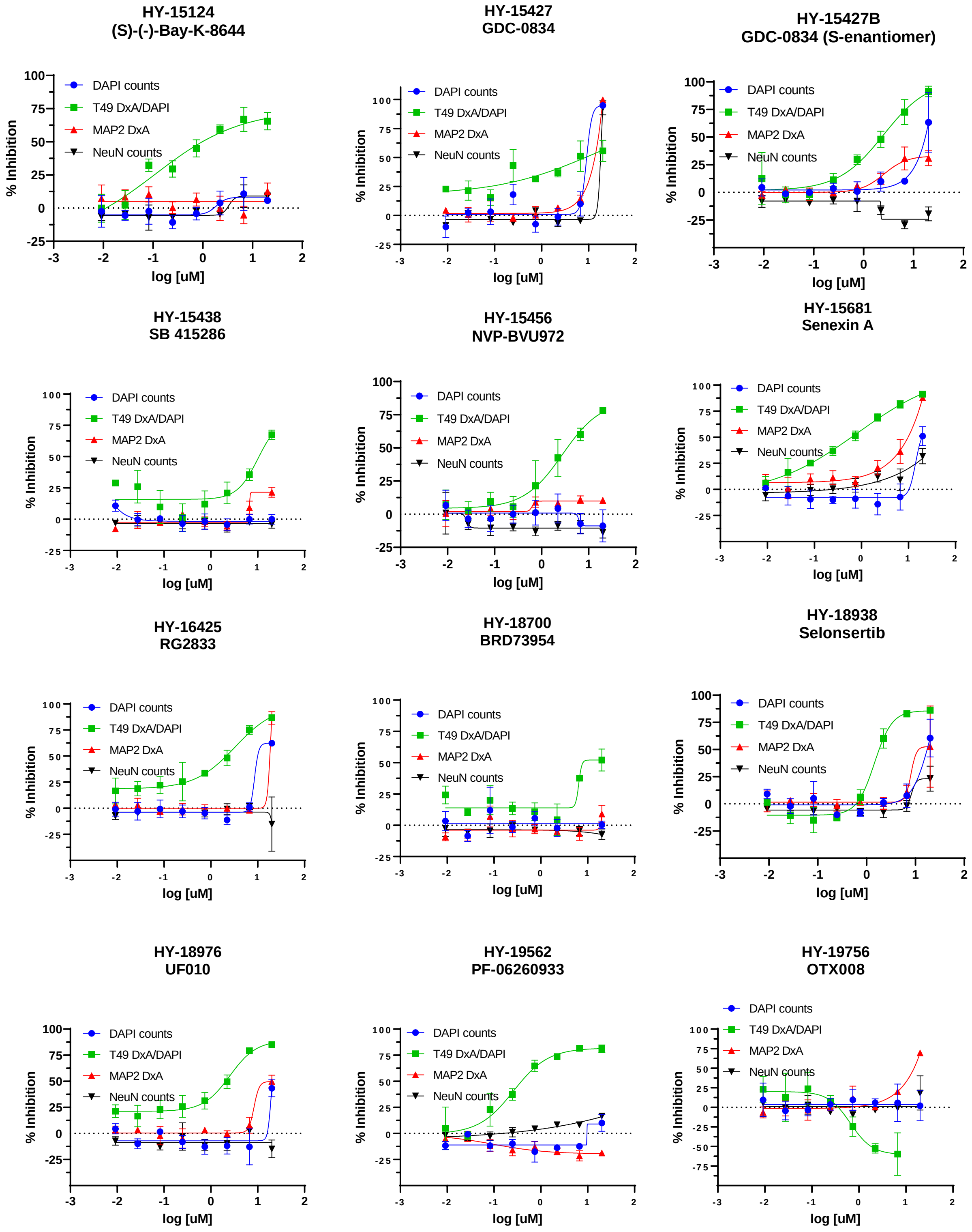

Figure S3. Continued

HY-19834
Fenebrutinib

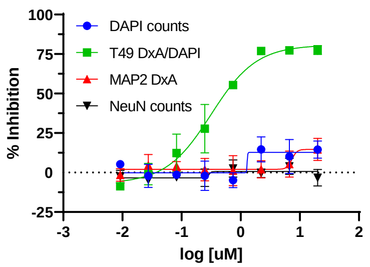

HY-59090
1-Azakenpaullone

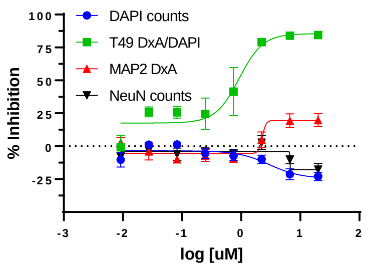

HY-70050C
Alosetron (Hydrochloride)

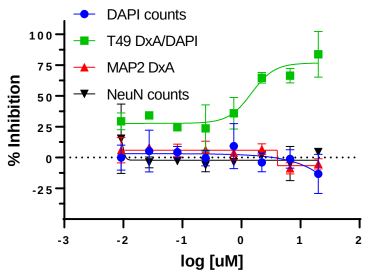

HY-76772
Cevimeline (hydrochloride hemihydrate)

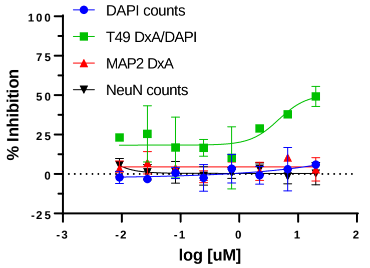

HY-79511
FAAH-IN-2

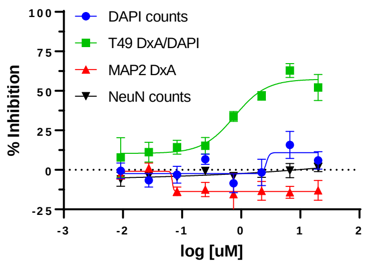

HY-100482
CPI-637

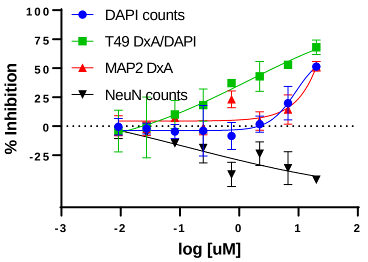

HY-100740C
(1α,1'S,4β)-Lanabecestat

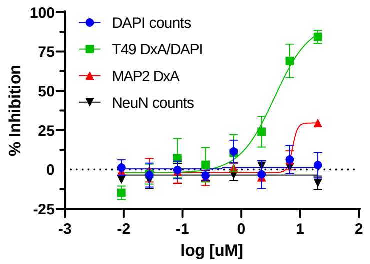

HY-100726
GNE-272

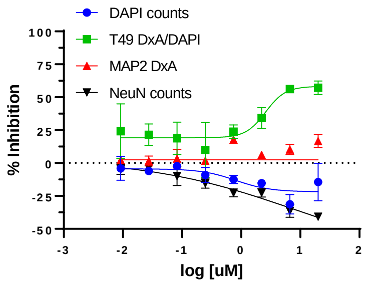

HY-101494
Temuterkib

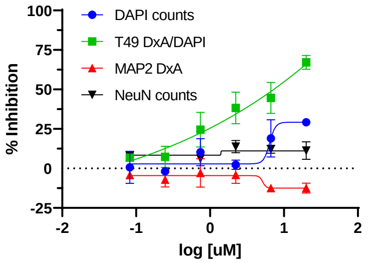

HY-101855
Anle138b

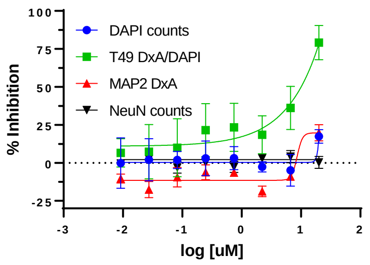

HY-107854
N-Acetyl-5-hydroxytryptamine

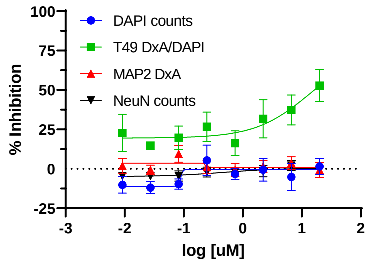

HY-112149
ZL0420

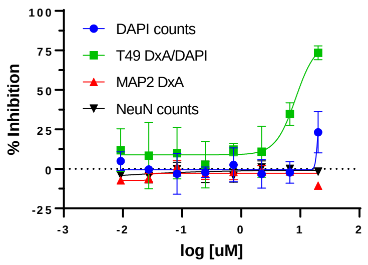

Figure S3. Continued

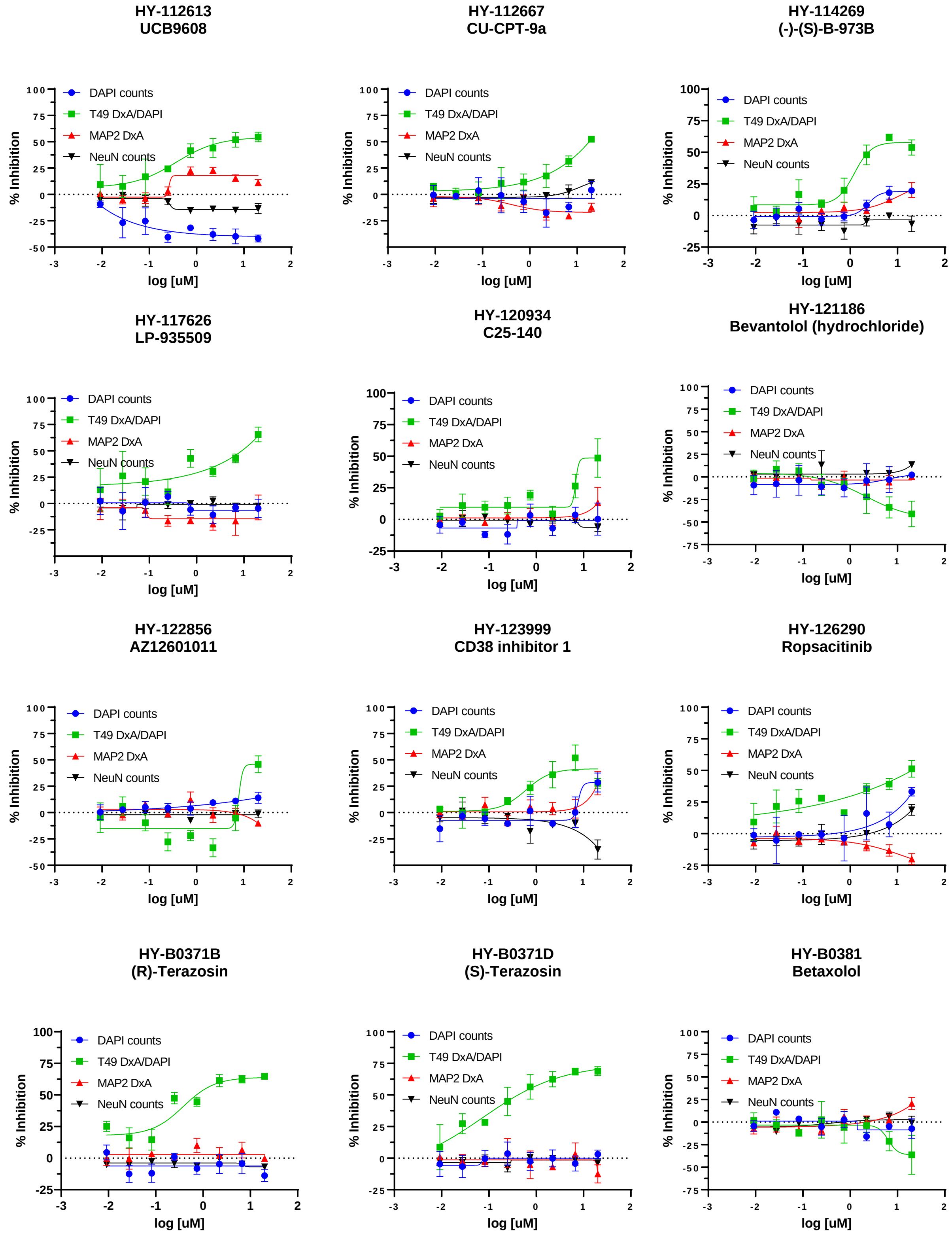

Figure S3. Continued

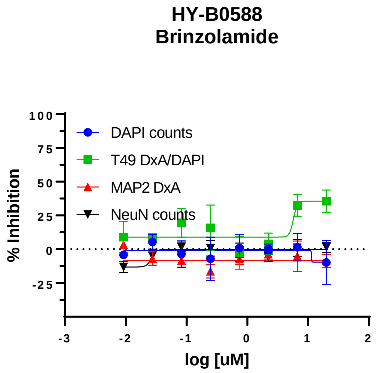

Figure S4. Testing of inhibitors of neuronal tau inclusions for effects on total endogenous soluble neuronal tau (blue). Compound effect on total neuronal homogenate protein levels are also plotted as a measure of toxicity (green).

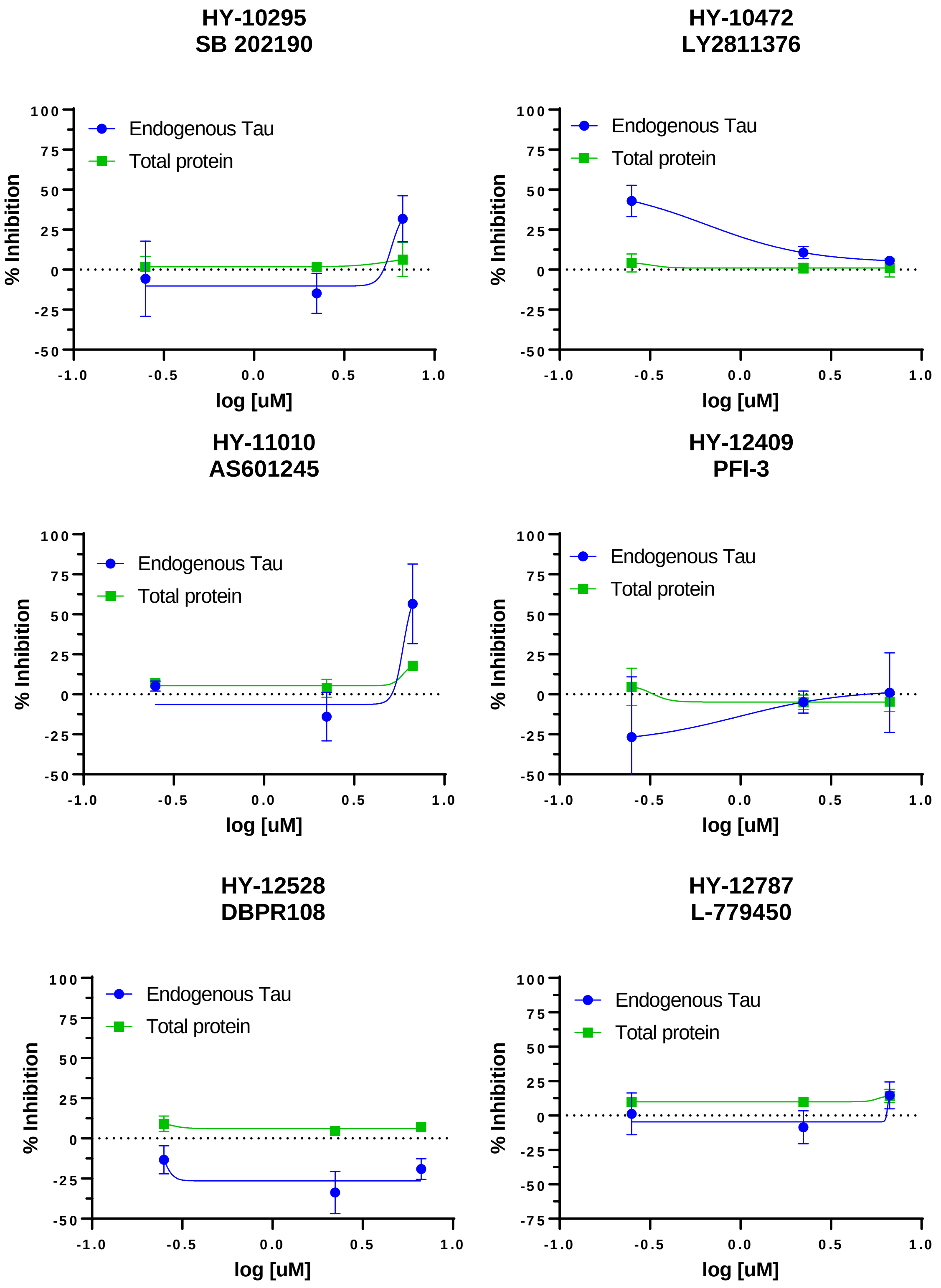

Figure S4. Continued

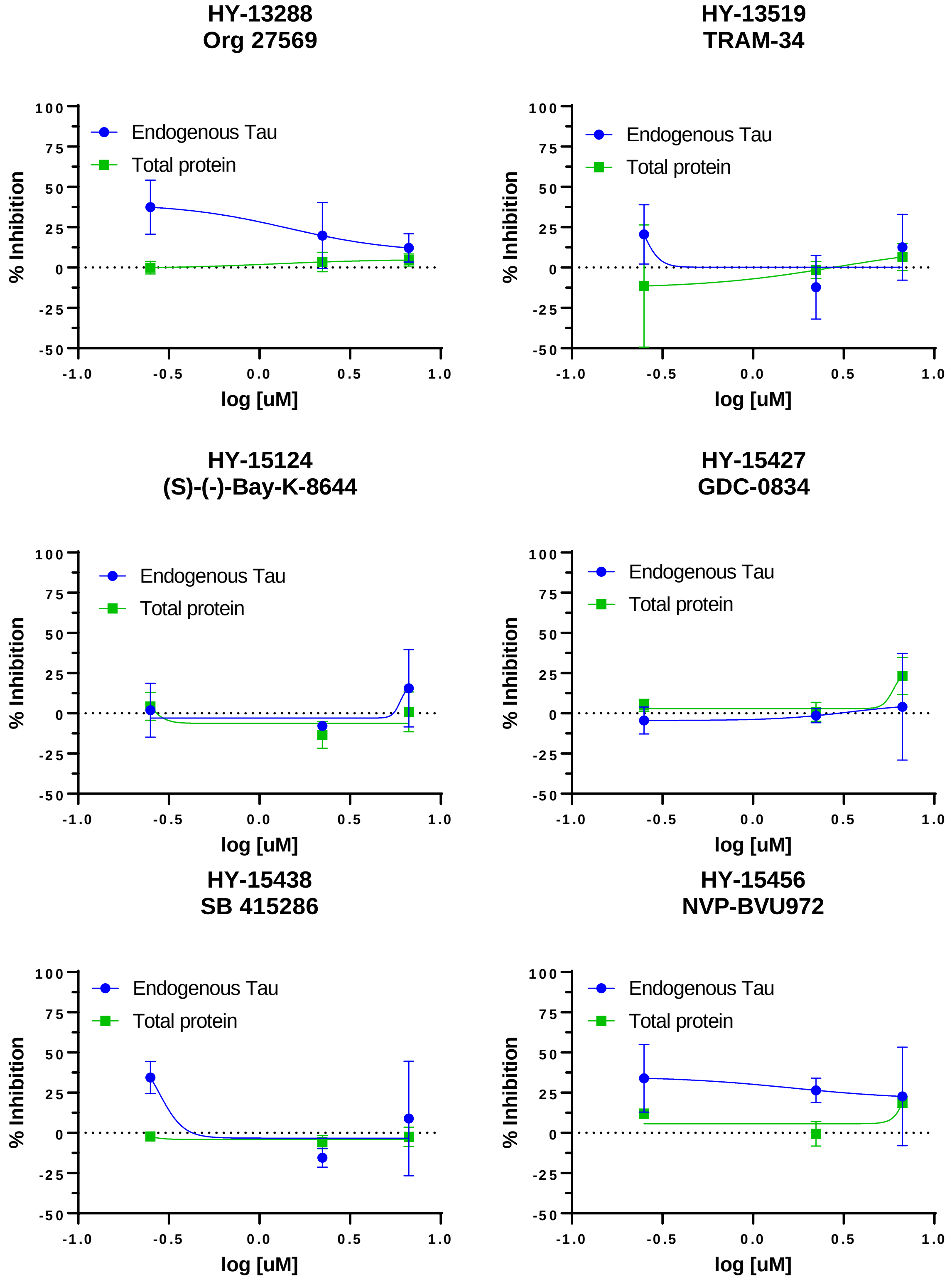

Figure S4. Continued

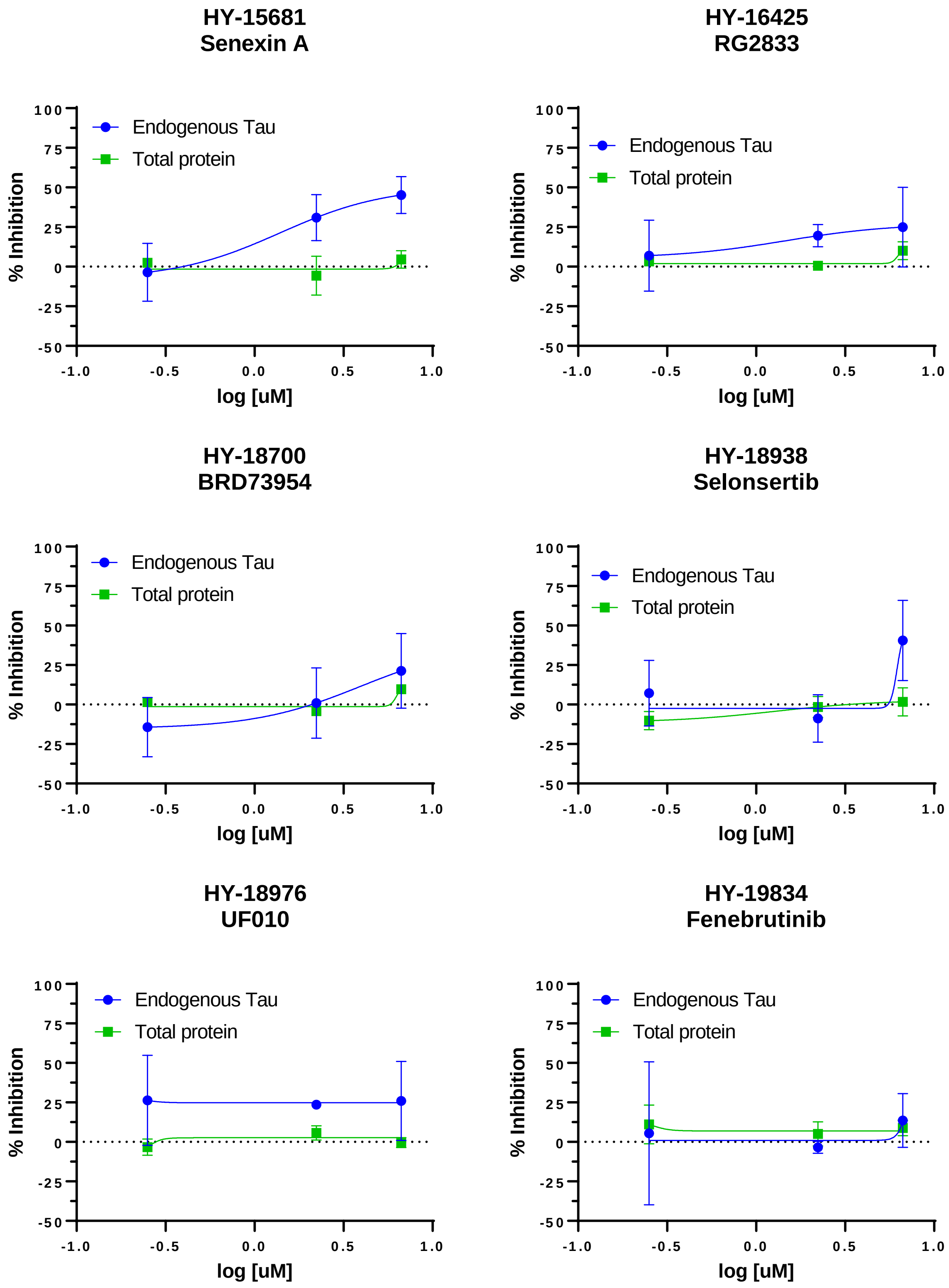

Figure S4. Continued

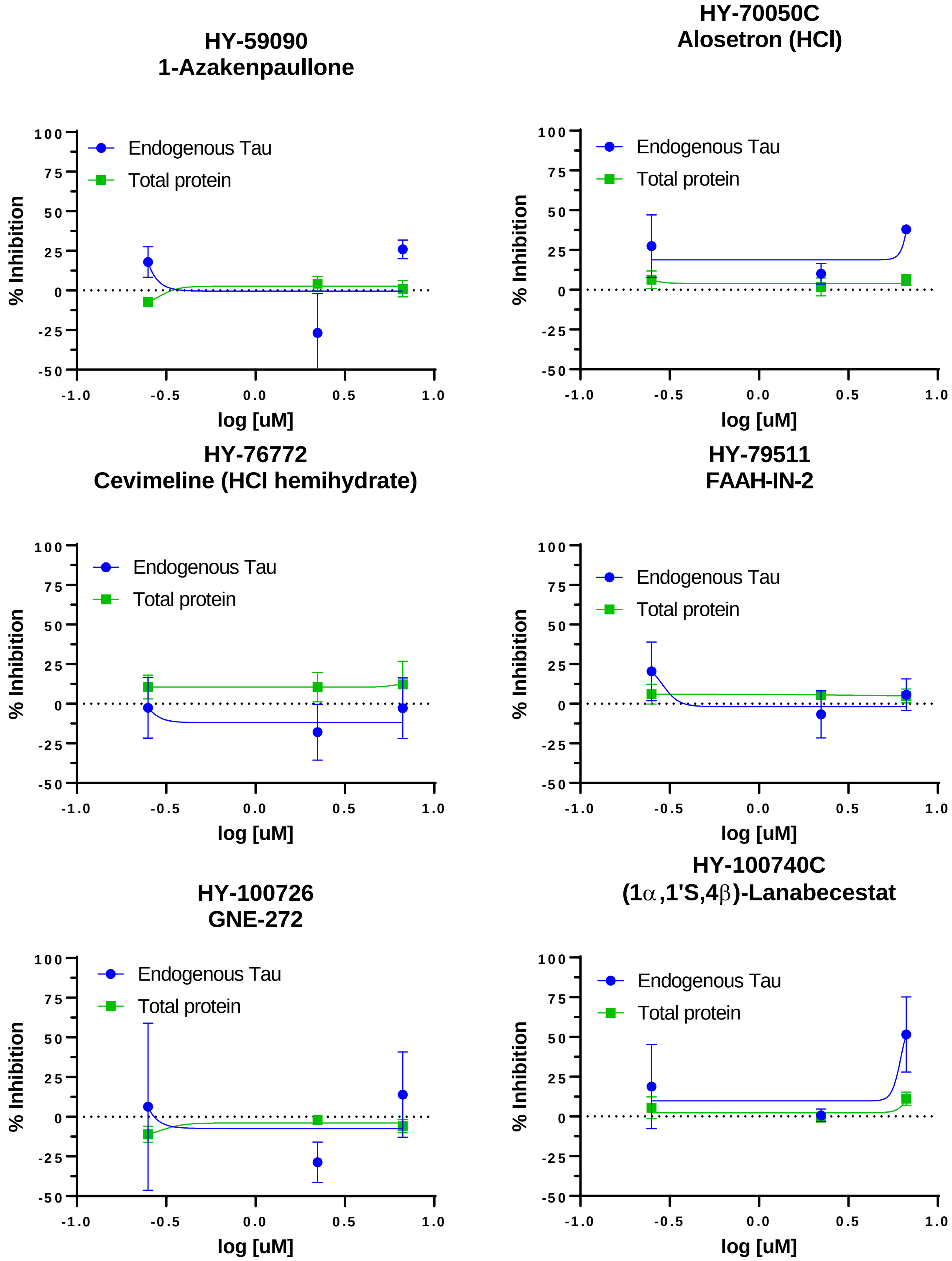

Figure S4. Continued

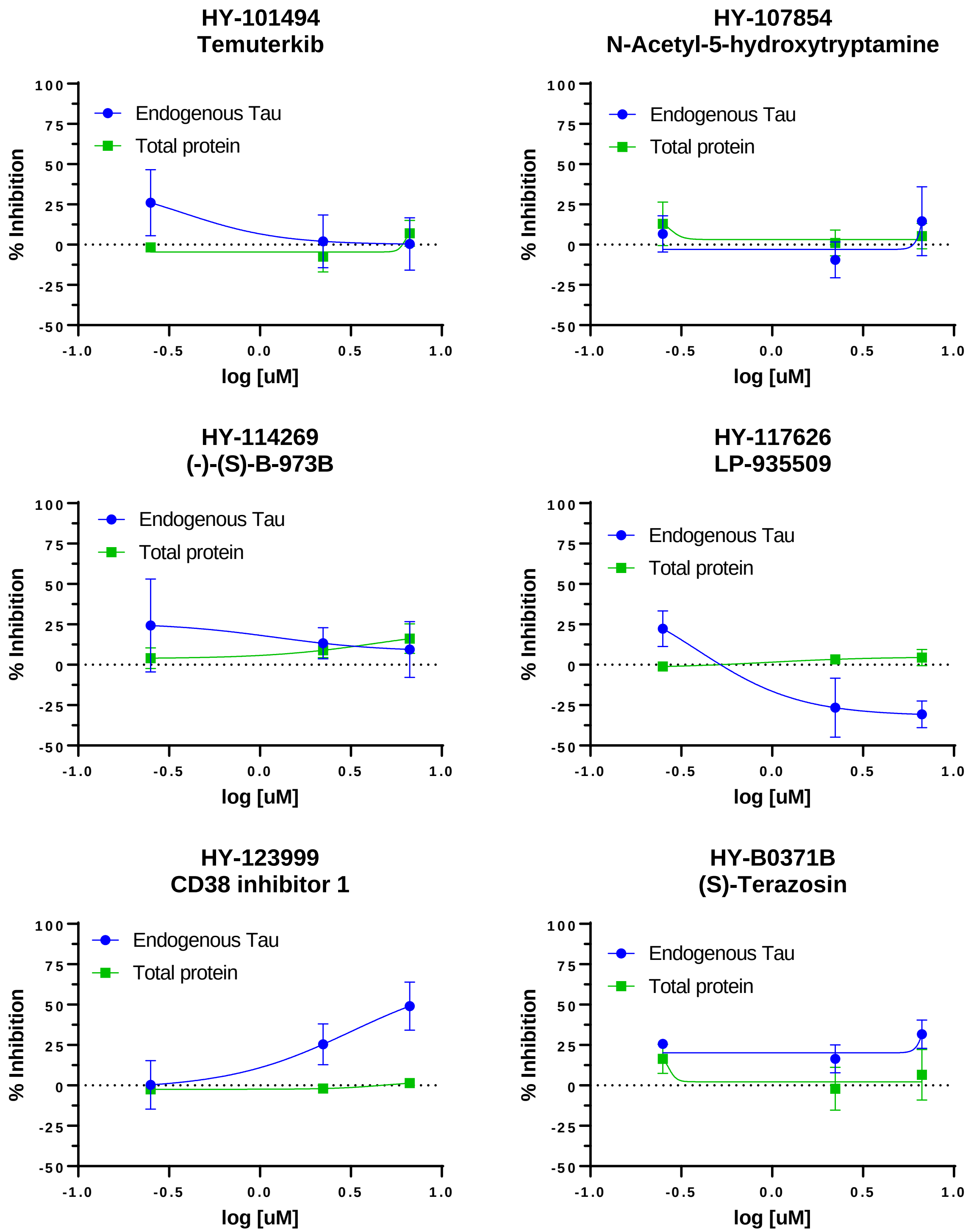

Figure S5. Representative immunoblots demonstrating that BACE1 and γ-secretase inhibitors reduce insoluble tau. **A**) Representative immunoblots of the insoluble pellet fraction (P) and soluble (S) fraction obtained after centrifugation of AD-tau-treated neuronal cultures treated with either vehicle (Veh) or the BACE1 inhibitors elenbecestat (EL) or LY2811376 (LY) at 3.3 µM. The blots were immunostained with the T49 antibody to detect rat tau and a GAPDH antibody for normalization. The pellet was re-suspended in ¼ the volume of the supernatant fraction, with equal volumes of both fractions loaded for analysis (20 µg total protein from the supernatant fractions). Each blot contains independent treatment samples. **B**) Representative immunoblot as above except comparing vehicle treatment to 1 µM of the γ-secretase inhibitor, E2012. Two vehicle- and three E2012-treated culture wells were included on this blot. These and additional blots with independent samples were used to generate the data in Figure 7A and 7B.

A.

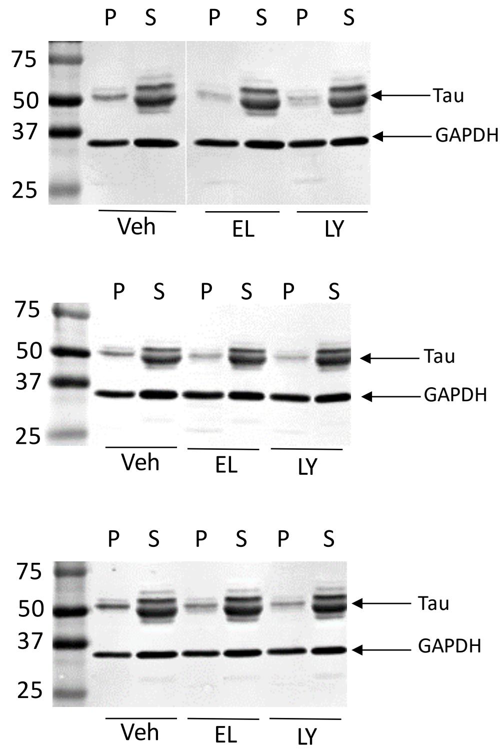

B.

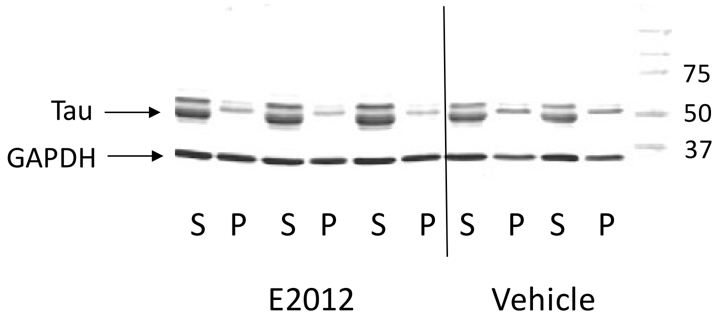

Figure S6. BACE1 and γ-secretase inhibitors do not alter soluble tau phosphorylation. **A**) Immunoblots of the soluble fractions obtained after centrifugation of AD-tau-treated neuronal cultures treated with either vehicle (Veh) or the BACE1 inhibitor LY2811376 at 3.3 µM, with the blots immunostained with the AT8, AT180 or PHF1 antibodies to detect phosphorylated tau. B) Similar blots are shown for AD-tau-treated neuronal cultures treated with either vehicle (Veh) or the γ-secretase inhibitor E2012 at 1 µM, with the AT8, AT180 or PHF1 antibodies used to detect phosphorylated tau. Blots were also immunostained with GAPDH antibody for normalization. Quantification is shown in Figure 7C and D.

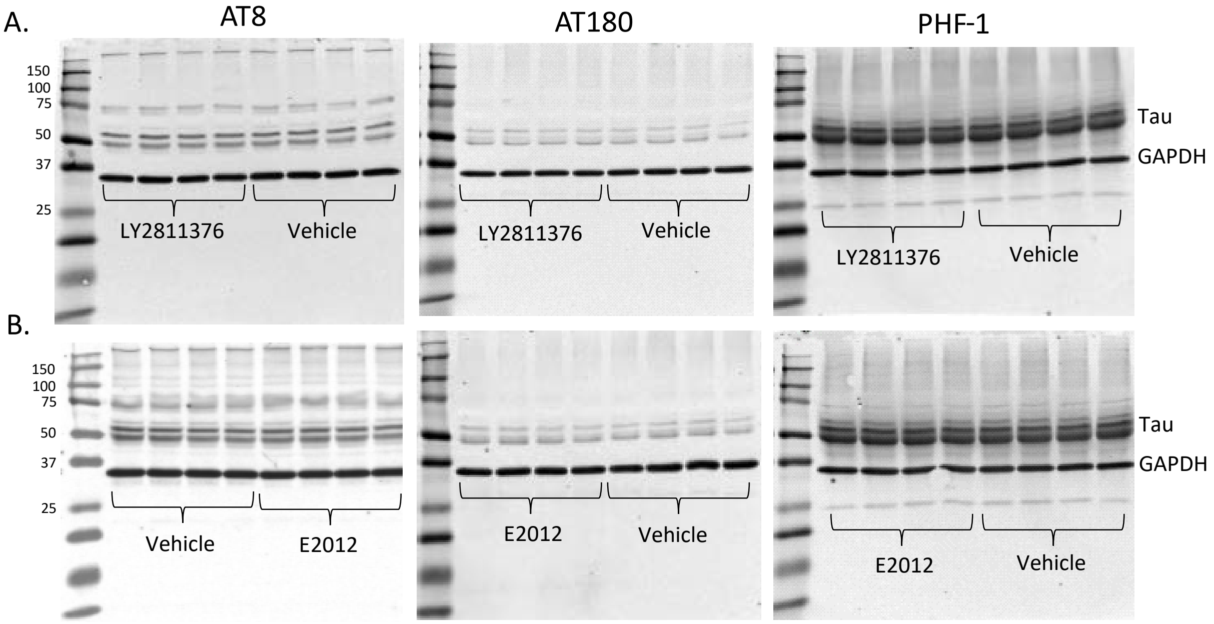

Figure S7. BACE1 and γ-secretase inhibitors cause a reduction of insoluble phosphorylated tau. **A**) Immunoblots of the insoluble pellet fractions obtained after centrifugation of AD-tau-treated neuronal cultures treated with either vehicle (Veh) or the BACE1 inhibitor LY2811376 at 3.3 µM, with the blots immunostained with the AT8 antibody to detect phosphorylated tau. B) Similar blots are shown for AD-tau-treated neuronal cultures treated with either vehicle (Veh) or the γ-secretase inhibitor E2012 at 1 µM, immunostained with the AT8 antibody. Blots were also immunostained with GAPDH antibody, with a fraction of GAPDH portioning into the pellet fraction. The pellet fractions were loaded as described in Figure S5. Quantification is shown in Figure 7E and F.

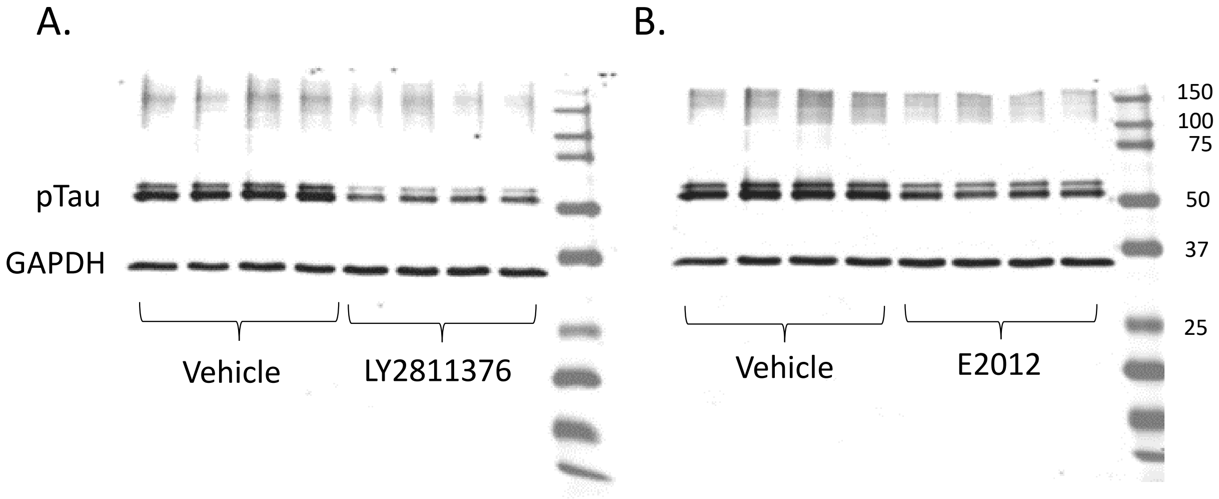

Supplement: Supporting Figures S1–S7 and Tables S1–S3 [file mmc1.pdf]
